# Guanxinshutong Alleviates Atherosclerosis by Suppressing Oxidative Stress and Proinflammation in ApoE^−/−^ Mice

**DOI:** 10.1155/2020/1219371

**Published:** 2020-09-16

**Authors:** Yingdong Lu, Yuchan Sun, Zhilin Jiang, Dandan Zhang, Hongchen Lin, Yi Qu, Chang Shang, Mingjing Zhao, Xiangning Cui

**Affiliations:** ^1^Department of Cardiology, Guang'anmen Hospital, China Academy of Chinese Medical Sciences, Beijing, China; ^2^The Key Laboratory of Chinese Internal Medicine of the Ministry of Education, Dongzhimen Hospital, Beijing University of Chinese Medicine, Beijing, China

## Abstract

Atherosclerosis (AS) is a chronic progressive disease related to dyslipidemia, inflammation, and oxidative stress. Guanxinshutong capsule (GXST), a traditional Chinese medicine, has been widely used in treating coronary atherosclerotic heart disease, while its mechanism actions on AS are still not to be well addressed. Our present study is aimed to examine the effect of GXST on AS and elucidate the multitarget mechanisms of GXST on AS. Network pharmacology analysis was employed to screen the multitarget mechanisms of GXST on AS. ApoE^−/−^ mice were used to validate these effects. Circulating lipid profile and oxidative stress-related factors were measured by the Elisa kit. Furthermore, the aortic trunk and aortic root were excised for oil red O staining, histopathological and immunohistochemical analysis. We first discovered that GXST was clued to exert synergistically antiatherosclerosis properties including lipid-lowering, anti-inflammation, and antioxidation through the computational prediction based on a network pharmacology simulation. Next, the validation experiments in atherosclerosis mice provided evidence that GXST significantly reduced atherosclerotic lesions, increased collagen deposition, and attenuated LV remodeling to some extent. Mechanistically, GXST modulated lipid profile, downregulated the level of inflammatory cytokines and NF-*κ*Bp65. GXST also reduced the activity of oxidative parameter MDA and upregulated the activities of antioxidant enzymes (SOD and GSH) compared with the AS model group. In conclusion, GXST intervention might attenuate atherosclerosis by mechanisms involving reducing lipid deposition, modulating oxidative stress and inflammatory responses, but a larger controlled trial is necessary for confirmation.

## 1. Introduction

Atherosclerosis (AS) is a chronic cardiovascular disease with high incidences of morbidity and mortality characterized by endothelial dysfunction, accumulation of cholesterol, recruitment of macrophage, and foam cells formation [1–3]. The pathophysiology of AS includes inflammatory response and oxidative stress as important factors. In this scenario, attenuating inflammation or ROS could exert a preventive role, thereby delaying or preventing the AS progression [4, 5].

Macrophages are the most prominent cellular contributors to the lesion's physical bulk [6] involved in the inflammatory response of AS. Decreasing the migration and adhesion of macrophages to the lesion area could effectively increase plaque stability. In addition, previous studies have shown that reducing the production of proinflammatory cytokines, such as IL-6, IL-1*β,* and TNF-*α*, and modulating the activity of nuclear factor kappa B (NF-*κ*B) could improve cardiovascular outcomes including AS [7, 8]. Moreover, accumulating evidence has demonstrated that oxidative stress caused by excessive reactive oxygen species (ROS) is closely associated with atherosclerosis progression [9, 10]. Previous data showed that patients with atherosclerotic cardiovascular disease (CVD) increased ROS in the vessel wall [11, 12]. The production of ROS by cells, such as macrophages and endothelial cell, is an important factor that influences smooth muscle cell migration, the homing of macrophage and inflammation, ox-LDL uptake, and fibrous cap stability [13]. Thus, attenuating oxidative stress and inflammation are maybe the critical approaches to improve AS considering their major role involved in AS.

Guanxinshutong capsule (GXST), a traditional Chinese medicine (TCM), is composed of five individual herbs according to the TCM theory of ‘Jun-Chen-Zuo-Shi', including Guangzao (*Choerospondiatis Fructus*), Danshen (*Radix Salviae*), Dingxiang (*Caryophylliflos*), Bingpian (*Borneolum Syntheticum*), and Tianzhuhuang (*Bamusaeconcretiosilicea*). Guangzao as a ‘Jun' (the monarch) herb exerts Qi promoting and blood activating effects; Danshen as a ‘Chen' (minister) herb is responsible for invigorating the blood to promote menstruation, dispelling block, and relieving pain; Dingxiang and Bingpian as ‘Zuo' (adjuvant) herbs are used for warming spleen and lowering the adverse flow of Qi and regulating the movement of Qi; Tianzhuhuang as a ‘Shi' (courier) herb contributes to clearing up the heat and resolving phlegm. In an ischemia and reperfusion (MI/R) injury study, GXST significantly decreased levels of TNF-*α*, IL-1*β*, and IL-6 and obviously inhibited NF-*κ*B activity in the MI/R model rat [14]. Furthermore, in a randomized clinical trial, GXST capsules are beneficial for the treatment of stable angina pectoris (SAP) patients [15]. It has also been shown that daily oral treatment of MI rats with GXST protects the heart against oxidative stress indicated by the increasing activities of total SOD, CATA, NOS and the levels of NO and GSH [16]. Although several pieces of evidence have supported GXST as anti-inflammation and antioxidant agent in CVD, few reports have evaluated whether those effects could modulate atherogenesis in vivo. Network biology aids the exploration of drug targets and identifies potential active ingredients in TCM research. Integrating network biology and polypharmacology promise an expanded opportunity for druggable targets. In this study, we intended to integrate network pharmacology techniques and pharmacological experiments to systematically identify the active components of GXST and explore potential mechanisms for the treatment of AS.

## 2. Materials and Methods

### 2.1. Screening the Effective Components of GXST

The active components of GXST were screened by the Traditional Chinese Medicine System Pharmacology Database (TCMSP, http://lsp.nwu.edu.cn/browse.php) and the Encyclopedia of Traditional Chinese Medicine (ETCM, http://www.nrc.ac.cn:9090/ETCM/), in which comprehensive information of the herbal ingredients such as components, pharmacokinetic parameter information, candidate target genes, and disease associated with the ingredients were provided [17, 18]. Absorption, distribution, metabolism, and excretion (ADME) are the critical contributors of pharmacokinetic parameters associated with bioactivities. In this study, oral bioavailability (OB, ≥30%), drug likeness (DL, ≥0.18) as suggested by TCMSP [19], and drug likeness grading more than 0.67 in ETCM were used as the included criteria of bioactive components in GXST [20].

### 2.2. Compound-Related Targets and AS-Related Targets Fishing

Three public databases including TCMSP, ETCM, and Drugbank [21] were applied to screen the compound-related targets. GeneCards (https://www.genecards.org/) and DisGeNET (https://www.disgenet.org/) database were used to collect the AS-related targets [22]. The keyword “atherosclerosis” was used, and the targets were human genes/proteins enrolled in our study. The standard gene names and UniProt ID of AS-related targets were obtained from UniprotKB database (https://www.uniprot.org/).

### 2.3. Gene Functions Annotation

Metascape (http://metascape.org/gp/index.html#/main/step1) was used to annotate the gene functions of compound-related targets. “*H. sapiens*” was selected for both the input species and the analysis species and *P* < 0.01 was set to analyze the function of the target. The biological processes related to atherosclerosis were screened, and target-functions were visualized and constructed by Cytoscape software.

### 2.4. Network Construction and Topological Analysis

The protein-protein interactions (PPI) network of the compound and AS-related cotargets were established from the STRING (https://string-db.org/) database which provides both experimental and predicted interaction information according to systematic coexpression analysis, detection of shared selective signals across genomes, and automated text-mining of scientific literature [23]. Confidence score ≥ 7 was selected in this study.

The central network analysis was performed by the topological method. Three topological parameters including degree centrality (DC), betweenness centrality (BC), and closeness centrality (CC) were calculated to evaluate the central attributes of the nodes in the network. In the GXST potential target-AS target network, DC ≥ 2×median DC, BC ≥ median BC, and CC ≥ median CC were used as the screening criteria to obtain the key targets.

### 2.5. GO and KEGG Pathway Enrichment Analysis

The online functional annotation and enrichment tool David (https://david.ncifcrf.gov/) was employed to perform the Gene Ontology (GO) and Kyoto Encyclopedia of Genes and Genomes (KEGG) pathway enrichment analysis [24]. *P* value <0.05 was considered statistically significant in GO terms and KEGG pathways.

### 2.6. Validating the GXST Functions by Animal Experiments

#### 2.6.1. Animals

ApoE^−/−^ mice on a C57BL/6 background and their wide-type littermates (male, eight weeks old) were purchased from Peking University School of Medicine (SCXK (Beijing) 2016-0012). Animals were maintained in groups under a 12 h/12 h light-dark cycle in a controlled environment at temperature 20 ± 2°C and humidity 50–60% with free access to food and water for 4 weeks. All experiment protocols involving animals were executed conforming to the Laboratory Animals of Institutional Animal Care. The protocol was approved by the Animal Ethics Committee of Guang'anmen Hospital, China Academy of Chinese Medical Sciences.

#### 2.6.2. Drug Administration

GXST capsules were purchased from Shaanxi Buchang Pharmaceutical (Z20020055). The ingredients of GXST capsule are Guangzao, Danshen, Dingxiang, Bingpian, and Tianzhuhuang. Atorvastatin calcium tablets were purchased from Pfizer Ireland Pharmaceuticals (National Drug Standard J20120050).

#### 2.6.3. Experimental Groups, Treatment, and Sample Preparation

After the adaptation period, wide-type mice were fed with a standard chow diet as a sham group (*n* = 16, 21.4 ± 0.3). ApoE^−/−^ mice (*n* = 48, 26.5 ± 0.4) were divided into three groups: (1) Model (*n* = 16), fed with a high-cholesterol diet (76.5% standard feed, 0.5% sodium cholate, 5% white sugar, 3% cholesterol, 10% lard, 5% egg yolk powder, provided by Beijing KeaoXieli Feed Co., Ltd., license number: SCXK (Beijing) 2014–0010.); (2) Atorvastatin (*n* = 16), fed with the above high-cholesterol diet and received atorvastatin treatment (5 mg·kg^−1^) by gavage feeding once daily; (3) GXST (*n* = 16), fed with the above high-cholesterol diet and received GXST treatment (0.18 g·kg^−1^) by gavage feeding once daily. Body weights were measured weekly. Although the study has been designed with equal group sizes, because of technical factors such as fight-related injury resulting in loss of animals, the group sizes become increasingly unequal (*n* = 13 for Sham group, *n* = 12 for Model group, *n* = 14 for Atorvastatin group, *n* = 13 for GXST group).

After 10 weeks and overnight fasting, animals were euthanized for blood and aorta collections. Serum was separated immediately after blood sampling by centrifugation at 3000 rpm for 15 min, and tissues (heart and aorta) were rapidly removed and frozen in liquid nitrogen.

#### 2.6.4. Serum Biochemical Assays

Serum concentrations of total cholesterol (TC), low density lipoprotein cholesterol (LDL-C), high density lipoprotein cholesterol (HDL-C), and triacylglycerols (TG) were determined by enzymatic colorimetric methods using commercially available kits. Murine serum TNF-*α*, IL-6 and oxidative stress-related factors (SOD, GSH, MDA) were detected by ELISA kits according to the manufacturer's introduction (BD Biosciences).

#### 2.6.5. Hematoxylin and Eosin (HE) and Masson's Trichrome Staining

The top halves of the hearts were obtained under stereoscopic observation, fixed by 4% paraformaldehyde, and embedded in paraffin. Briefly, every consecutive section (6 *μ*m thick) throughout the aortic root area (300 *μ*m) was stained with hematoxylin/eosin. One of every five sections (10 sections/mouse) and the leftover sections were stained with Masson's trichrome staining. The lesion and collagen deposition areas were quantified with Image-Pro Plus 6.0 software (NIH, Bethesda, MD, USA).

#### 2.6.6. Atherosclerotic Lesions Evaluation

Atherosclerotic lesion severity was assessed in the aortas as previously described [25]. In brief, animals were perfused with PBS and 4% paraformaldehyde. Then, the entire mouse aorta was carefully dissected from the arch to the iliac bifurcation to remove adventitious, washed in PBS, and the aorta was opened longitudinally, stained with Oil red O (Sigma-Aldrich, USA), and pinned out under an inverted microscope. Digital images were analyzed with Image-Pro Plus version 6.0 and the extent of the lesion area is expressed as the percentage of the total area of the aorta covered by the lesions.

#### 2.6.7. Immunohistochemistry

The paraffin-embedded aorta sinus sections (4 *μ*m) were deparaffinized for citrate or EDTA antigen retrieval according to the manufacturer's instructions, blocked with hydrogen peroxide (H_2_O_2_, 3%), and incubated with different primary antibodies: CD68 (Abcam, ab125212, 1 : 500), TNF-*α* (Abcam, ab66579, 1 : 200), IL-6 (Abcam, ab9324, 1 : 300), NF-*κ*B (Santa Cruz, sc-8414, 1 : 500), Nrf2 (Abcam, ab137550, 1 : 300), and HO-1 (Abcam, ab13243, 1 : 500) for 60-minute incubation at 37°C. Finally, the sections were washed again with PBS for DAB colorimetry. The positive sections were quantified by Image-Pro Plus 6.0 software (NIH, Bethesda, MD, USA).

#### 2.6.8. Western Blots

Total proteins of the aorta were extracted using RIPA agents and quantified with a BCA protein kit to determine protein levels. Briefly, the samples were separated by a 12% sodium dodecylsulfate-polyacrylamide gel electrophoresis and transferred to a poly-vinylidene difluoride filter membrane. Membranes were blocked in Tris-buffered saline (PH7.5) containing 0.05% Tween-20 (1 × TBST) and 5% skimmed milk for 2 h, then probed overnight at 4°C with an antibody against NF-*κ*Bp65, Nrf2, and HO-1, respectively. Membranes were washed with 1 × TBST for 10 min and incubated for 2 h with horseradish peroxidase-conjugated secondary antibody. Lastly, immunoreactive bands were visualized by chemiluminescence and quantified by densitometry using a Quantity One Analysis Software (Bio-Rad).

#### 2.6.9. Statistical Analysis

All procedures were performed in triplicate. All data were presented as the mean ± standard deviation (SD). An independent-samples *t*-test, one-way analysis of variance (ANOVA) was conducted to evaluate the one-way layout data. *P* values less than 0.05 were considered significant. All analyses were performed using GraphPad Prism6.0 (GraphPad Software, Inc., La Jolla, CA, USA).

## 3. Results

### 3.1. Herb-Compound-Target Network of GXST

**Figure 1:**
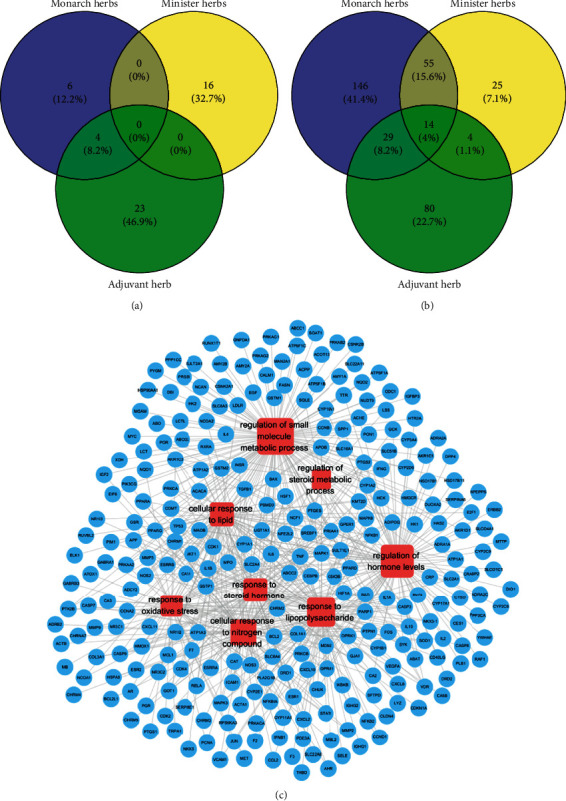
Herb-compound-target network of GXST, (a) distribution of active compounds among the herbs, (b) distribution of potential targets among the herbs, (c) network of the herb-compounds targets function. Blue nodes represent targets. Red nodes represent the enrichment of biological function and the size of nodes is proportional to degree centrality by topology analysis.

A total of 49 active compounds and 353 targets after excluding the duplicates were identified in GXST from TCMSP and ETCM database. Ten active compounds, 244 targets from the “monarch herb”, 16 active compounds, 98 targets from the “minister herb” and 27 active components, and 127 targets from the “adjuvant and courier herbs” were identified (Figures 1(a) and 1(b)). The detailed 49 components and 353 targets information are shown in Supplementary Table S1 and Supplementary Table S2. Total of 106 targets for atherosclerosis were acquired from GeneCards and DisGeNET databases after removing redundancies which are shown in Supplementary Table S3. To enrich the biological function of the 353 targets, Metascape (http://metascape.org/gp/index.html#/main/step1) was used to annotate gene functions which uncovered that 353 targets were enriched in cellular response to lipid, oxidative stress, lipopolysaccharide, nitrogen compound, metabolic process, and steroid hormone response (Figure 1(c)).

### 3.2. Pathway Enrichment Analysis

**Figure 2:**
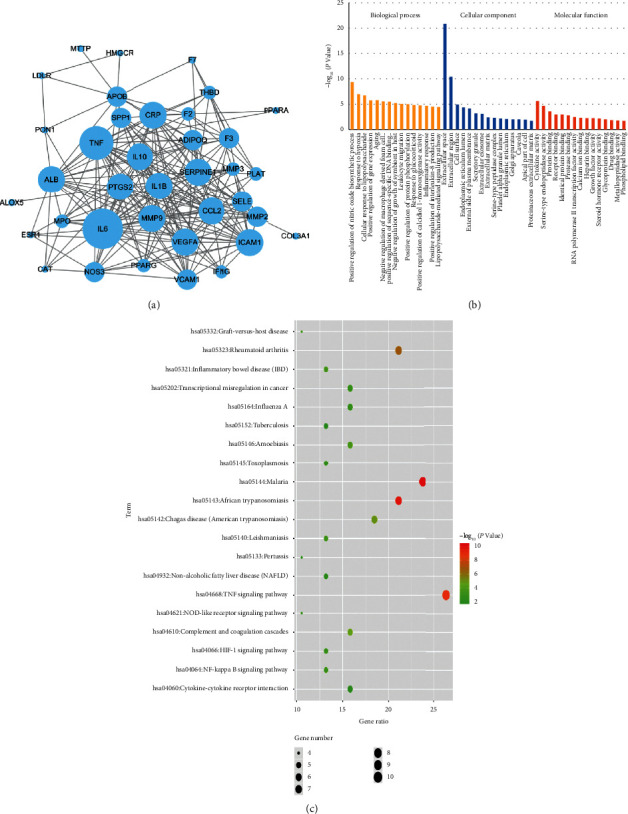
Pathway enrichment analysis, (a) network of the 38 shared targets between GXST potential targets and AS targets, (b) GO enrichment analysis for 38 key targets, (c) KEGG enrichment analysis for 38 key targets. Blue nodes represent targets and the size of nodes is proportional to degree centrality by topology analysis.

Totally, 38 cotargets were collected as potential therapeutic targets of GXST against AS after 353 potential targets of the 49 compounds in GXST intersected with 106 candidate targets for AS. There were 445 PPIs among these 38 cotargets with high confidence scores (≥7) (Figure 2(a)). Topology analysis indicated that TNF-*α*, IL-1*β*, IL-6, IL-10, PTGS2, MMP9, VEGFA, CCL2, CRP, and ICAM1 were the top 10 shared targets based on degree centrality. GO analysis showed that the top 15 biological processes ranked by their LogP were enriched to the regulation of inflammatory response, leukocyte migration, macrophage derived foam cell differentiation, and nitric oxide biosynthetic process (Figure 2(b)). Furthermore, KEGG pathway enrichment analysis showed that 38 cotargets contributed to 158 pathways (*P* < 0.05), and the first 20 pathways were closely related to the pathogenesis of AS (Figure 2(c)).

### 3.3. Validation the Protective Effects of GXST against AS Progression

#### 3.3.1. GXST Reduces the Lesion Area and Changes the Plaque Composition

**Figure 3:**
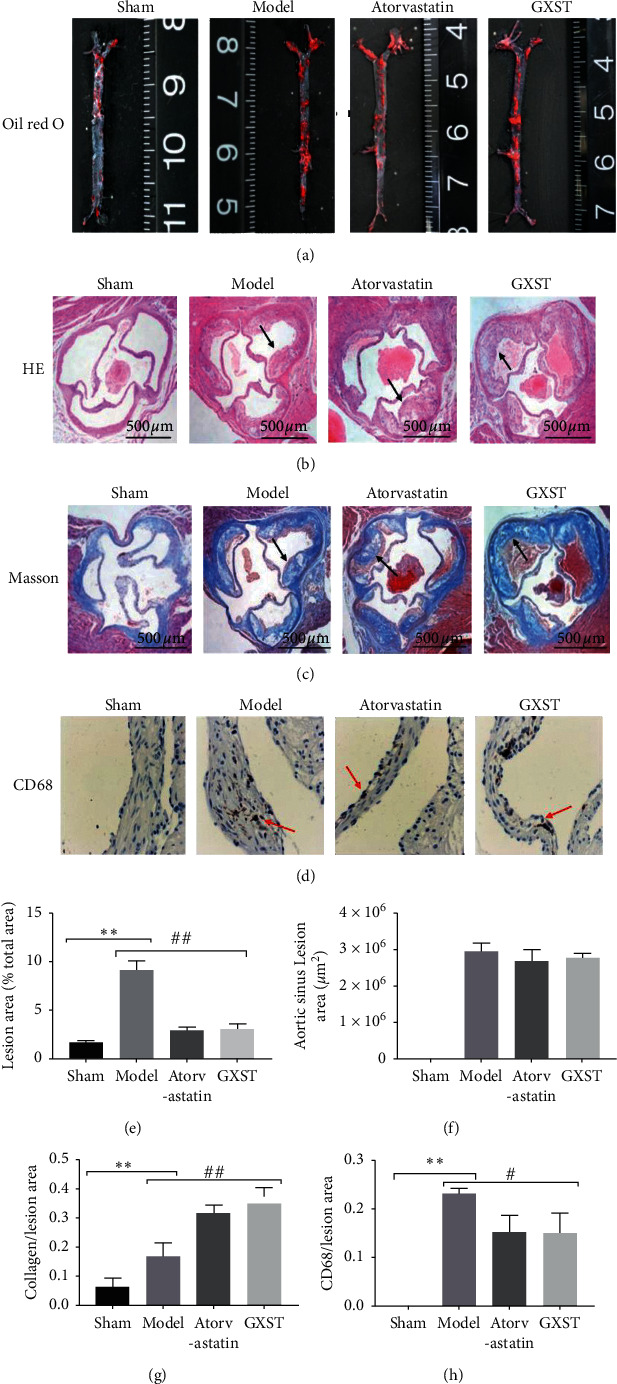
GXST reduces the lesion area and plaque composition, (a) representative images of oil red O-stained aortic trunk in each group (*n* = 3), (b) representative images of HE-stained aortic sinus sections from Sham, model, GXST, and atorvastatin (*n* = 5), (c) representative images of Masson-stained aortic sinus sections from Sham, model, GXST, and atorvastatin (*n* = 5), (d) representative photomicrographs images of aortic root sections stained with CD68 in each group (*n* = 3), (e) the size of the aortic trunk lesion was calculated as the percentage of lesion area in each group (*n* = 5), (f) quantitative analysis of the lesion size root in each group (*n* = 8), (g) quantitative analysis of the extracellular matrix in each group (*n* = 8), (h) quantitative analysis of CD68 in each group (*n* = 5). ^*∗*^*P* < 0.05 showed a significant difference compared with the Sham. ^#^*P* < 0.05 showed a significant difference compared with the model, ^*∗*^*P* < 0.05, ^*∗∗*^*P* < 0.01, ^*∗∗∗*^*P* < 0.001; ^#^*P* < 0.05, ^##^*P* < 0.01, ^###^*P* < 0.001).

ApoE^−/−^ mice were applied as AS model in our study, and the knockdown efficiency of ApoE expression in mice was confirmed by PCR (Supplementary Table S4, Supplementary Figure 1). In order to verify whether GXST has the protective effect against AS progression, we analyzed the lesion area of the aortic trunk by oil red O staining and aortic sinus by HE staining. We also studied the plaque composition including collagen and macrophages via Masson and immunohistochemical staining (CD68). In our study, atherosclerosis in the aorta was reduced in 48% of the GXST group, mainly in the aortic arch (Figures 3(a) and 3(e)). However, the aortic sinus of all groups presented similar lesions in the intermediate stage except the Sham, with the presence of a fibrous cap and areas of deposition of cholesterol crystals (Figures 3(b) and 3(f)). Although the total lesion in aortic sinus was similar between groups, GXST significantly presented lower macrophage infiltration (CD68) and increased collagen deposition, suggesting a more stable fibrous cap (Figures 3(c), 3(d), 3(g), and 3(h)). In order to further evaluate the therapeutic effects of GXST on AS, we assessed the left ventricular cardiomyocyte morphology and interstitial fibrosis. As shown in the H&E staining image, the GXST group was demonstrated to significantly improve the myocardial shape, arrangement, and nuclear morphology in the LV wall compared to that from the model group mice (Supplementary Figure 4). Meanwhile, the Model group had a significantly higher degree of fibrosis than that of the Sham, while GXST treatment reversed such a trend (Supplementary Figure 4).

#### 3.3.2. GXST Attenuates Plasma Lipid Profiles in ApoE^−/−^ Mice

**Figure 4:**
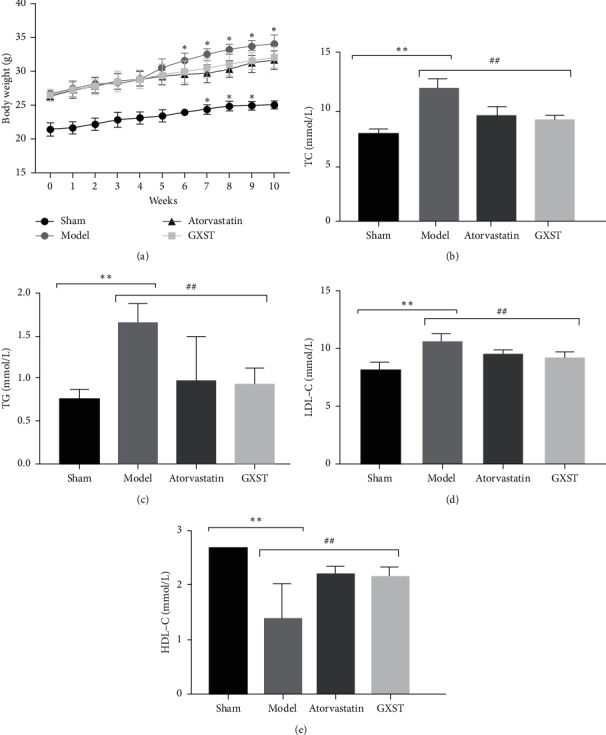
Effects of GXST on body weight and serum lipid levels in ApoE^−/−^ mice, (a) body weight in the four groups was compared every week. The effects of GXST treatment on plasma cholesterol levels: TC (b), TG (c), LDL-C (d), and HDL-C (e); Sham (normal saline, *n* = 13); Model (ApoE^−/−^ male mice, *n* = 12); ATF (Model group fed on atorvastatin, *n* = 14); GXST (Model group fed on GXST capsule, *n* = 13). ^*∗*^*P* < 0.05 showed a significant difference compared with the Sham. ^#^*P* < 0.05 showed a significant difference compared with the model, ^*∗*^*P* < 0.05, ^*∗∗*^*P* < 0.01, ^*∗∗∗*^*P* < 0.001; ^#^*P* < 0.05, ^##^*P* < 0.01, ^###^*P* < 0.001.

The results from the network pharmacological analysis suggested that the biological function of GXST on AS was enriched in the cellular response to lipid, oxidative stress, and lipopolysaccharide. Herein, we first evaluated the level of weight gain and plasma lipid profiles. A high-fat diet with 3% cholesterol for 10 weeks in the Model, Atorvastatin, and GXST groups resulted in a significant increase (36.1%, 26.5%, 27.8%) in body weight compared with the Sham group without a high-fat diet (Figure 4(a)). GXST treatment did not significantly inhibit high-fat diet-induced weight gain compared to the Model group after 10 weeks treatment, while GXST prominently induced the loss of weight during from 7 to 9 weeks, and atorvastatin significantly decreased the weight gain from the fifth-week treatment (Figure 4(a)). In addition, serum biochemical parameters of mice including TC, TG, LDL-C, and HDL-C were determined (Figures 4(b)–4(e)). Compared to the Model, GXST markedly decreased the high-fat diet-induced elevation in TC, TG, and LDL-C, whereas it increased the level of HDL-C. As expected, atorvastatin also improved the serum lipid profile.

#### 3.3.3. GXST Attenuates the Inflammatory Response

**Figure 5:**
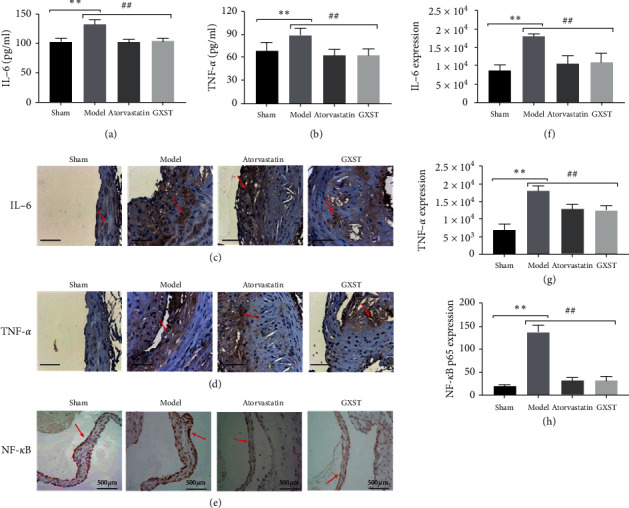
GXST attenuates the inflammatory response, (a and b) Blood IL-6 and TNF-*α* levels (*n* = 8), (c–e) representative images of IL-6, TNF-*α,* and NF-*κ*B in the aortic sinus (*n* = 3), (f–h) quantitative analysis of the IL-6, TNF-*α*, and NF-*κ*B expression in the aortic sinus (*n* = 5). ^*∗*^*P* < 0.05 showed a significant difference compared with the Sham. ^#^*P* < 0.05 showed a significant difference compared with the model, ^*∗*^*P* < 0.05, ^*∗∗*^*P* < 0.01, ^*∗∗∗*^*P* < 0.001; ^#^*P* < 0.05, ^##^*P* < 0.01, ^###^*P* < 0.001).

Topology analysis uncovered the top 10 shared targets between GXST-compounds related and AS targets. Herein, IL-6 and TNF-*α* concentration in plasma and plaque lesion were significantly decreased in the GXST group (Figures 5(a)–5(d), 5(f), and 5(g). In addition, consistent with the results of the above proinflammatory factor, the expression level of NF-*κ*B in the aortic lesion was significantly reduced (Figures 5(e) and 5(h), Supplementary Figure 5).

#### 3.3.4. GXST Attenuated Reactive Oxidative Stress-Associated Factor

**Figure 6:**
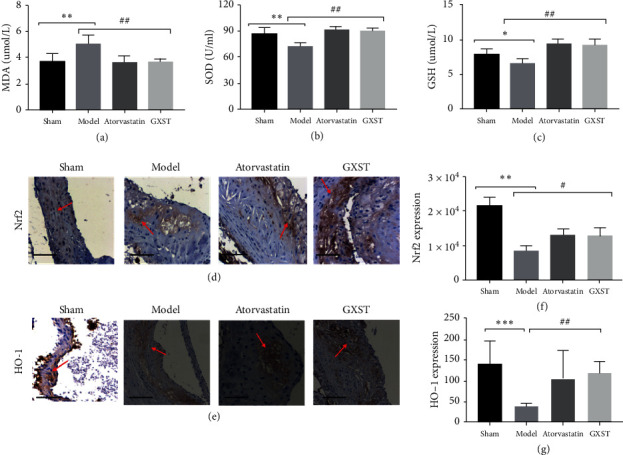
GXST attenuated oxidative stress, (a–c) blood MDA, GSH, and SOD levels (*n* = 8), (d-e) representative images of Nrf2 and HO-1 in the aortic sinus (*n* = 3). (f-g) Quantitative analysis of Nrf2 and HO-1 in the aortic sinus (*n* = 5). ^*∗*^*P* < 0.05 showed a significant difference compared with the Sham. ^#^*P* < 0.05 showed a significant difference compared with the model, ^*∗*^*P* < 0.05, ^*∗∗*^*P* < 0.01, ^*∗∗∗*^*P* < 0.001; ^#^*P* < 0.05, ^##^*P* < 0.01, ^###^*P* < 0.001.

According to the GO analysis and KEGG pathway enrichment, the compounds of GXST may also target oxidative stress. Malondialdehyde (MDA), an important ROS marker of lipid peroxidation, increases in AS progression [26]. Glutathione (GSH) and superoxide dismutase (SOD) are important components of the antioxidant defense mechanism that antagonise the effect of oxidative stress [27]. Analyzing the level of MDA, GSH, and SOD in plasma, we found that GXST significantly decreased MDA while it increased GSH and SOD compared to Model group (Figures 6(a)–6(c)). Nrf2 is a member of the CNC-b ZIP transcription factor family. On physiological conditions, Nrf2 binds with the negative regulatory factor Keap1 in the cytoplasm. Under ROS stress, Nrf2 decouples with Keap1 enters the nucleus and combines with SOD and detoxification enzyme HO-1 to complete the protective role of antioxidative stress. In our study, the Model group significantly decreased the expression of Nrf2 and HO-1, while GXST restored them (Figures 6(d)–6(g), Supplementary Figure 5).

## 4. Discussion

AS is a chronic cardiovascular disease whose pathophysiology includes lipid metabolism dysfunction, inflammation, and oxidative stress. The effect of GXST on AS has not been illuminated before the present study. However, its anti-inflammatory, antioxidative effects in other cardiovascular diseases are seen. In this context, a total of 49 active compounds in GXST and 353 compound-related targets, and 106 AS-related targets were identified from the public databases. Among these targets, 38 targets were shared between compound-related and AS-related targets, implicating the possible anti-AS action of GXST. From the PPI network, the top 10 targets were enriched in the inflammatory response and lipid deposition. GO terms and KEGG pathway enriched the function of GXST on cellular response to lipid, inflammation, and oxidative stress. The preventive effect of GXST against AS was verified in ApoE^−/−^ mice. Altogether, these results indicated that GXST modulated atherosclerosis by regulating lipids, inhibiting inflammatory activity, and ROS. The putative active ingredients and multitarget mechanisms of GXST in the treatment of AS were elucidated in the present study for the first time, which provided theoretic evidence for the clinical application of GXST on AS treatment.

ApoE is a glycoprotein with a structural component of all lipoprotein particles except low-density lipoproteins, and it is synthesized mainly in the liver and brain and plays a vital role in lipids metabolism [28]. It has been confirmed that ApoE deficiency contributes to the accumulation of cholesterol-rich remnants in plasma and thus induced AS [29]. ApoE^−/−^ mouse is widely used in the research for AS as it can manifest pathological features of human AS [30–32]. Thus, ApoE knockout mouse was established with high lipid diet in our present study.

From the perspective of traditional Chinese medicine (TCM), the fundamental problem arteriosclerosis caused by hyperlipidemia is the deficiency of Spleen Qi, which leads to the “stagnation” of phlegm. “Stasis” and “Phlegm” block in the vascular wall and these are consistent with the pathology changes of vascular. GXST, a combination of the traditional herb and Mongolian medicine, is well-known to play an important role in promoting blood and QI circulation, removing blood stasis and phlegm, and relieving chest pain.

Analyzing the atherosclerotic lesions of GXST and Sham groups, we noticed that the lesion area in the aorta tree was reduced, while it was not modified in the aortic sinus after GXST treatment. Such discrepancy may reflect the different stages of AS development observed at those sites. As a previous study described, atherosclerotic lesions occur faster in the aortic sinus due to the turbulent blood flow that occurs caused by the faster heart rate in mice [33], while the lesions develop more slowly in the aorta. CD68 is a heavily glycosylated glycoprotein that is highly expressed in macrophages and other mononuclear phagocytes. Traditionally, CD68 is exploited as a valuable cytochemical marker to immune stain monocyte/macrophages in the histochemical analysis. In addition, in the AS progression, monocytes can infiltrate plaques, become activated, and develop into macrophages that can accumulate in the vascular wall. These cells engulf high levels of lipids depending on the variety of scavenger receptors (SR) on the membrane of macrophages such as CD68 and eventually form foam cells that accelerate atherosclerotic plaque formation. In our study, GXST significantly decreased the infiltration of macrophages marked by CD68 in the aortic sinus, although the total lesion in the aortic sinus was similar between GXST and Model groups. Moreover, augmented collagen deposition was indicated by Masson staining. Altogether, GXST treatment changed the plaque composition, presenting lower macrophage infiltration and augmented collagen deposition, suggesting a more stable fibrous cap. These results demonstrate the benefits of GXST in stabilizing the fibrous cap and modulating foam cell formation in AS progression. Furthermore, AS is a chronic disease of the arterial wall which is related to life-threatening complications such as myocardial infarction. Our data demonstrate that, in addition to beneficial stable plaques effects, GXST moderately improves LV remodeling indicated by well-ordered cardiomyocyte and lowered interstitial fibrosis. This kind of target organ protection by GXST is at least partially dependent on the attenuation of AS in ApoE^−/−^ mice.

Based on our network pharmacology analysis of GXST on AS, diverse underlying mechanisms including lipid-lowing, inflammatory response, and oxidative stress might be associated with the modulation of AS progression. Hypercholesterolemia is being the risk factor sufficiently to cause atherosclerotic, even in the absence of other cardiovascular risk factors [27]. Evidence from clinical trials indicates that a high level of circulating LDL-C level as well as low levels of HDL-C are associated with CVD risk, and the reduction of LDL-C and excessive HDL-C may decrease the incidence of atherosclerotic diseases [34, 35]. In addition, a large number of studies have shown that hypertriglyceridemia contributes to the development and progression of atherosclerosis [36]. Therefore, it is very important to modulate the lipid profile in attenuating AS progression. In our study, we found that GXST treatment markedly reduced the APOE^−/−^ mice body weight from the 7th to 9th week treatment. Similarly, GXST significantly decreased the plasma concentration of TG, TC, and LDL-C and increased the HDL-C levels.

For over a decade now, GXST has an anti-inflammatory effect. A recent study reported that GXST decreased levels of TNF-*α*, IL-1*β*, IL-6 and obviously inhibited the NF-*κ*B pathway [14]. In order to confirm GXST-related anti-inflammatory response associated with reduced atherosclerotic lesions and macrophage infiltration in the aortic sinus, we examined several inflammatory markers of atherosclerosis in plasma and aorta. TNF-*α* has a central role in controlling inflammatory processes and atherosclerosis development independent of plasma cholesterol levels. Inhibitiing TNF-*α* expression or capturing TNF-*α* biological action may inhibit advanced lesion formation [37]. IL-6 induced by TNF-*α* is regarded as a proinflammatory cytokine in AS [38]. Our results showed that GXST treatment reduced the levels of TNF-*α* and IL-6 in plasma and aortic lesion. Nuclear factor kappa B (NF-*κ*B), a major transcription factor, regulates the inflammatory response and participates in the AS progression [39, 40]. NF-*κ*B is a key transcription factor induced by oxLDL and inflammatory cytokines, which, in turn, induces the expression of proinflammatory cytokines such as TNF-*α* and IL-6. The activity of NF-*κ*B depends on its activation through its translocation into the nucleus. It is well known that oxLDL or proinflammatory cytokine activates the p65/p50 dimer of phosphorylated IkB which can migrate to the nucleus and induce gene transcription [41]. In our present study, the GXST supplement decreased the expression of NF-*κ*Bp65 in the aortic sinus indicated by immunohistochemistry staining and western blots. Thus, we suggest that GXST could decrease TNF-*α* and IL-6 secretion partly by reducing NF-*κ*B activation. These results implied that GXST could inhibit the inflammatory reaction via downregulating the expression of proinflammatory cytokines including IL-6 and TNF-*α* mediated by NF-*κ*B.

Several studies have suggested that oxidative stress also participates in atherosclerotic development and plaque instability [9, 42]. ROS can be generated as metabolic by-products by nearly all cell types. Decreasing ROS levels is a useful strategy for the treatment of AS [43]. GXST has been reported to protect the heart against oxidative stress indicated by the increasing activities of total SOD, NOS and the levels of NO and GSH [16]. The present study found that in ApoE^−/−^ mice treated with GXST, the SOD (converts the highly reactive ROS to more stable) and GST (reduces the levels of ROS) were increased. Moreover, a defense mechanisms called nuclear erythroid factor 2- related factor 2 (Nrf2)/heme oxygenase-1 (HO-1) pathway was reported to participate in attenuating oxidative stress [44]. Nrf2 could dissociate with kelch-like ECH-associatedprotein1 (Keap1, the cytosolic inhibitor of Nrf2), translocate to the nucleus and activate the downstream antioxidant enzymes such as SOD2 and HO-1, and ultimately degrade ROS production and reduce oxidative injury. The present study has indicated that inhibition of HO-1 expression in hyperlipidemic rabbits leads to higher levels of circulating lipid profile and greater atherosclerotic lesions [45]. Herein, GXST treatment was found to increase Nrf2 and HO-1 expression associated with ROS degradation. Overall, these results highlight that GXST ameliorates oxidative stress by decreasing ROS generation and increasing ROS degradation.

## 5. Conclusions

In conclusion, the present study predicted drug-target-disease interactions and mutitarget mechanisms of GXST on AS progression by network pharmacology strategy and validated the attenuation effect of GXST against AS in vivo. This study showed that the effect of GXST on stabilizing the fibrous cap and modulating foam cell formation was associated with attenuating dyslipidemia, inflammation and oxidative stress.

## Data Availability

The data used to support the findings of this study are available from the corresponding author upon request.

## Supplementary Materials

Supplementary Materials Supplementary Table S1. Characteristics of active compounds in GXST. This file contains the herbs of GXST and herbs-associated compounds, molecular formula, molecular weight (MW), oral bioavailability (OB), drug-likeness, PubchemID, and SMILES. Supplementary Table S2. The potential targets of the compounds in GXST. This file contains the herbs' compounds and compound-associated targets, Uniprot ID and Gene Code. Supplementary Table S3. AS-related target. This file includes the gene name, target name, Uniprot ID, and the source of the database of AS-related target. Supplementary Table S4. Primer sequences for PCR. Supplementary Figure 1. The knock down efficiency of ApoE expression in mice. Total DNA was extracted from the heart of C57BL/6J wide type (liver) and ApoE^−/−^ mice (liver, brain, heart, and aorta) according to the manufacturers's instructions. The DNA was used as a template to perform PCR with TaKaRa PCR Amplification Kit (TaKaRa Biotechnology) both in wide type (150 bp) and APOE deficiency mice (250 bp). M: DL 2000 marker; N: negative control; P: positive control; 1–8: ApoE^−/−^ mice applied in our study; 9-10: C57BL/6J wide type (liver, liver). Supplementary Figure 2. The lower magnitude for HE, Masson staining (magnification: ×20), and CD68 of the aortic sinus (magnification: ×40). Supplementary Figure 3. The lower magnitude for IL-6, TNF-*α* and NF-*κ*B of the aortic sinus (magnification: ×40). Supplementary Figure.4. GXST moderately improves LV remodeling. (A) Representative images of HE staining of left ventricular wall (*n* = 3). (B) Representative images of Masson staining of left ventricular wall (*n* = 3). (C) Quantitative analysis of the extracellular matrix in each group (*n* = 5). ^*∗*^*P* < 0.05 showed a significant difference compared with the Sham. ^#^*P* < 0.05 showed a significant difference compared with the Model, ^*∗∗*^*P* < 0.01, ^##^*P* < 0.01. Supplementary Figure 5. The effects of GXST on the protein levels of inflammatory and oxidative stress factors in the aorta. (A) Western blot results of aorta NF-*κ*Bp65, Nrf2, and HO-1 expression. (B) Quantitative analysis of the expression level in each group (*n* = 3). ^*∗*^*P* < 0.05 showed a significant difference compared with the Sham. ^#^*P* < 0.05 showed a significant difference compared with the Model. ^##^*P* < 0.01.
