# Supplementary material for: Guanxinshutong Alleviates Atherosclerosis by Suppressing Oxidative Stress and Proinflammation in ApoE−/− Mice
Source: Evid Based Complement Alternat Med. 2020 Sep 16;2020:1219371. doi: 10.1155/2020/1219371 (PMC7519182; doi:10.1155/2020/1219371)
Supplement: Supplementary Materials — Supplementary Table S1. Characteristics of active compounds in GXST. This file contains the herbs of GXST and herbs-associated compounds, molecular formula, molecular weight (MW), oral bioavailability (OB), drug-likeness, PubchemID, and SMILES. Supplementary Table S2. The potential targets of the compounds in GXST. This file contains the herbs' compounds and compound-associated targets, Uniprot ID and Gene Code. Supplementary Table S3. AS-related target. This file includes the gene name, target name, Uniprot ID, and the source of the database of AS-related target. Supplementary Table S4. Primer sequences for PCR. Supplementary Figure 1. The knock down efficiency of ApoE expression in mice. Total DNA was extracted from the heart of C57BL/6J wide type (liver) and ApoE−/− mice (liver, brain, heart, and aorta) according to the manufacturers's instructions. The DNA was used as a template to perform PCR with TaKaRa PCR Amplification Kit (TaKaRa Biotechnology) both in wide type (150 bp) and APOE deficiency mice (250 bp). M: DL 2000 marker; N: negative control; P: positive control; 1–8: ApoE−/− mice applied in our study; 9-10: C57BL/6J wide type (liver, liver). Supplementary Figure 2. The lower magnitude for HE, Masson staining (magnification: ×20), and CD68 of the aortic sinus (magnification: ×40). Supplementary Figure 3. The lower magnitude for IL-6, TNF-α and NF-κB of the aortic sinus (magnification: ×40). Supplementary Figure.4. GXST moderately improves LV remodeling. (A) Representative images of HE staining of left ventricular wall (n = 3). (B) Representative images of Masson staining of left ventricular wall (n = 3). (C) Quantitative analysis of the extracellular matrix in each group (n = 5). ∗P < 0.05 showed a significant difference compared with the Sham. #P < 0.05 showed a significant difference compared with the Model, ∗∗P < 0.01, ##P < 0.01. Supplementary Figure 5. The effects of GXST on the protein levels of inflammatory and oxidative stress factors in the aorta [file 1219371.f1.zip › 1219371.f1/Supplementary Table S1.docx]

Supplementary Table S1. Characteristics of active compounds in GXST

|  | **Herbs** | **Compounds** | **Molecular formula** | **MW** | **OB%** | **DL** | **PubchemID** | **SMILES** |
| --- | --- | --- | --- | --- | --- | --- | --- | --- |
| Monarch herb | choerospondiatts fructus(guang zao) | Stepharine | C18H19NO3 | 297.35 | 31.55 | 0.906 | 98455 | COC1=C(C2=C3C(CC24C=CC(=O)C=C4)NCCC3=C1)OC |
| Monarch herb | choerospondiatts fructus(guang zao) | Camphor | C10H16O | 152.26 | 31.68 | 0.519 | 444294 | CC1(C2CCC1(C(=O)C2)C)C |
| Monarch herb | choerospondiatts fructus(guang zao) | Glucose | C6H12O6 | 180.16 | 50.38 | 0.305 | 5793 | C(C1C(C(C(C(O1)O)O)O)O)O |
| Monarch herb | choerospondiatts fructus(guang zao) | Jujuboside B | C52H84O21 | 1045.2 | 58.67 | 0.095 | 102004532 | CC1C(C(C(C(O1)OC2C(C(COC2OC3CCC4(C5CCC6C7C(CC(OC78CC6(C5(CCC4C3(C)C)C)CO8)C=C(C)C)(C)O)C)O)OC9C(C(C(C(O9)CO)O)O)OC1C(C(C(CO1)O)O)O)O)O)O |
| Monarch herb | choerospondiatts fructus(guang zao) | (2R)-5,7-dihydroxy-2-(4-hydroxyphenyl)chroman-4-one | C15H12O5 | 272.27 | 42.36 | 0.21 | 667495 | C1C(OC2=CC(=CC(=C2C1=O)O)O)C3=CC=C(C=C3)O |
| Monarch herb | choerospondiatts fructus(guang zao) | (-)-taxifolin | C15H12O7 | 304.27 | 60.51 | 0.27 | 712316 | C1=CC(=C(C=C1C2C(C(=O)C3=C(C=C(C=C3O2)O)O)O)O)O |
| Monarch herb | choerospondiatts fructus(guang zao) | beta-sitosterol | C29H50O | 414.79 | 36.91 | 0.75 | 222284 | CCC(CCC(C)C1CCC2C1(CCC3C2CC=C4C3(CCC(C4)O)C)C)C(C)C |
| Monarch herb | choerospondiatts fructus(guang zao) | kaempferol | C15H10O6 | 286.25 | 41.88 | 0.24 | 5280863 | C1=CC(=CC=C1C2=C(C(=O)C3=C(C=C(C=C3O2)O)O)O)O |
| Monarch herb | choerospondiatts fructus(guang zao) | naringenin | C15H12O5 | 272.27 | 59.29 | 0.21 | 439246 | C1C(OC2=CC(=CC(=C2C1=O)O)O)C3=CC=C(C=C3)O |
| Monarch herb | choerospondiatts fructus(guang zao) | quercetin | C15H10O7 | 302.25 | 46.43 | 0.28 | 5280343 | C1=CC(=C(C=C1C2=C(C(=O)C3=C(C=C(C=C3O2)O)O)O)O)O |
| Minister herb | Radix Salviae(dan shen) | Salvilenone | C20H20O2 | 292.37 | 38 | 0.776 | 389885 | CC1=C2C=CC3=C4C2=C(C=C1)C(=O)C(=C4OC3(C)C)C(C)C |
| Minister herb | Radix Salviae(dan shen) | 3-beta-Hydroxymethyllenetanshiquinone | C18H14O4 | 294.30 | 41 | 0.759 | 5318290 | CC1=COC2=C1C(=O)C(=O)C3=C2C=CC4=C3CCC(C4=C)O |
| Minister herb | Radix Salviae(dan shen) | 5,6-dihydroxy-7-isopropyl-1,1-dimethyl-2,3-dihydrophenanthren-4-one | C19H22O3 | 298.41 | 33.77 | 0.29 | [11011966](http://pubchem.ncbi.nlm.nih.gov/summary/summary.cgi?cid=11011966) | CC(C)C1=C(C(=C2C(=C1)C=CC3=C2C(=O)CCC3(C)C)O)O |
| Minister herb | Radix Salviae(dan shen) | 4-methylenemiltirone | C18H18O2 | 266.36 | 34.35 | 0.23 | 14609851 | CC(C)C1=CC2=C(C3=C(C=C2)C(=C)CCC3)C(=O)C1=O |
| Minister herb | Radix Salviae(dan shen) | NSC 122421 | C20H28O2 | 300.48 | 34.49 | 0.28 | [94162](http://pubchem.ncbi.nlm.nih.gov/summary/summary.cgi?cid=94162) | CC(C)C1=C(C=C2C(=C1)C(=O)CC3C2(CCCC3(C)C)C)O |
| Minister herb | Radix Salviae(dan shen) | 1-methyl-8,9-dihydro-7H-naphtho[5,6-g]benzofuran-6,10,11-trione | C17H12O4 | 280.29 | 34.72 | 0.37 | 10062187 | CC1=COC2=C1C(=O)C(=O)C3=C2C=CC4=C3CCCC4=O |
| Minister herb | Radix Salviae(dan shen) | sugiol | C20H28O2 | 300.44 | 38 | 0.791 | 94162 | CC(C)C1=C(C=C2C(=C1)C(=O)CC3C2(CCCC3(C)C)C)O |
| Minister herb | Radix Salviae(dan shen) | luteolin | C15H10O6 | 286.24 | 35 | 0.598 | 5280445 | C1=CC(=C(C=C1C2=CC(=O)C3=C(C=C(C=C3O2)O)O)O)O |
| Minister herb | Radix Salviae(dan shen) | poriferast-5-en-3beta-ol | C29H50O | 414.79 | 36.91 | 0.75 | [457801](http://pubchem.ncbi.nlm.nih.gov/summary/summary.cgi?cid=457801) | CCC(CCC(C)C1CCC2C1(CCC3C2CC=C4C3(CCC(C4)O)C)C)C(C)C |
| Minister herb | Radix Salviae(dan shen) | Methylenetanshinquinone | C18H14O3 | 278.32 | 37.07 | 0.36 | [105118](http://pubchem.ncbi.nlm.nih.gov/summary/summary.cgi?cid=105118) | CC1=COC2=C1C(=O)C(=O)C3=C2C=CC4=C3CCCC4=C |
| Minister herb | Radix Salviae(dan shen) | dihydrotanshinlactone | C18H14O3 | 266.31 | 38.68 | 0.32 | 11778300 | CC1COC2=C1C(=O)C3=C(C2=O)C4=CC=CC(=C4C=C3)C |
| Minister herb | Radix Salviae(dan shen) | 1,2,5,6-tetrahydrotanshinone | C18H16O3 | 280.34 | 38.75 | 0.36 | 124416 | CC1COC2=C1C(=O)C(=O)C3=C2C=CC4=C3CCC=C4C |
| Minister herb | Radix Salviae(dan shen) | Miltirone | C19H22O2 | 282.41 | 38.76 | 0.25 | [160142](http://pubchem.ncbi.nlm.nih.gov/summary/summary.cgi?cid=160142) | CC(C)C1=CC2=C(C3=C(C=C2)C(CCC3)(C)C)C(=O)C1=O |
| Minister herb | Radix Salviae(dan shen) | dan-shexinkum d | C21H20O4 | 336.38 | 38.88 | 0.869 | 127172 | CC1COC2=C(C1(C)CO)C(=O)C3=C(C2=O)C4=CC=CC(=C4C=C3)C |
| Minister herb | Radix Salviae(dan shen) | neocryptotanshinone ii | C17H18O3 | 270.35 | 39.46 | 0.23 | [5320066](http://pubchem.ncbi.nlm.nih.gov/summary/summary.cgi?cid=5320066) | CC1=C(C2=C(C3=C(C=C2)C(CCC3)(C)C)C(=O)C1=O)O |
| Minister herb | Radix Salviae(dan shen) | microstegiol | C20H26O2 | 298.46 | 39.61 | 0.28 | 403772 | CC1=C2CCCC(C3(C2=C(C=C1)C=C(C3=O)C(C)C)O)(C)C |
| Adjuvant and guide herbs | caryophylli flos (ding xiang) | Benzaldehyde | C_6_H_5_CHO | 106.12 | 32.63 | 0.497 | 240 | C1=CC=C(C=C1)C=O |
| Adjuvant and guide herbs | caryophylli flos (ding xiang) | Vanillin | C8H8O3 | 152.15 | 52 | 0.65 | 1183 | COC1=C(C=CC(=C1)C=O)O |
| Adjuvant and guide herbs | caryophylli flos (ding xiang) | Γ-Caryophyllene | C14H22 | 190.32 | 64 | 0.5 | 16667688 | CC1=CCC(=C)C2CC(C2CC1)(C)C |
| Adjuvant and guide herbs | caryophylli flos (ding xiang) | Eugenol | C10H12O2 | 164.22 | 56.24 | 0.04 | [3314](http://pubchem.ncbi.nlm.nih.gov/summary/summary.cgi?cid=3314) | COC1=C(C=CC(=C1)CC=C)O |
| Adjuvant and guide herbs | caryophylli flos (ding xiang) | Benzyl Acetate | C9H10O2 | 150.17 | 40.79 | 0.612 | 8785 | CC(=O)OCC1=CC=CC=C1 |
| Adjuvant and guide herbs | caryophylli flos (ding xiang) | Chavicol | C9H10O | 134.18 | 44.19 | 0.616 | 68148 | C=CCC1=CC=C(C=C1)O |
| Adjuvant and guide herbs | caryophylli flos (ding xiang) | Eugenitin | C12H12O4 | 220.24 | 36.39 | 0.787 | 3083581 | CC1=CC(=O)C2=C(C(=C(C=C2O1)OC)C)O |
| Adjuvant and guide herbs | caryophylli flos (ding xiang) | Eugenone | C13H16O5 | 252.29 | 38.7 | 0.566 | 5317271 | CC(=O)CC(=O)C1=C(C=C(C=C1OC)OC)OC |
| Adjuvant and guide herbs | caryophylli flos (ding xiang) | Isoengelitin | C21H22O10 | 434.43 | 34.65 | 0.7 | 101937309 | CC1C(C(C(C(O1)OC2C(OC3=CC(=CC(=C3C2=O)O)O)C4=CC=C(C=C4)O)O)O)O |
| Adjuvant and guide herbs | caryophylli flos (ding xiang) | Isoeugenitin | C12H12O4 | 220.22 | 53.45 | 0.787 | 12310980 | CC1=CC(=O)C2=C(O1)C(=C(C=C2O)OC)C |
| Adjuvant and guide herbs | caryophylli flos (ding xiang) | Isoeugenitol | C11H10O4 | 206.19 | 48.7 | 0.68 | 5318562 | CC1=CC(=O)C2=C(C=C(C(=C2O1)C)O)O |
| Adjuvant and guide herbs | caryophylli flos (ding xiang) | M-Methoxybenzaldehyde | C8H8O2 | 136.16 | 48.8 | 0.577 | 11569 | COC1=CC=CC(=C1)C=O |
| Adjuvant and guide herbs | caryophylli flos (ding xiang) | Methyl-N-Pentyl Ketone | C7H14O | 114.21 | 46.56 | 0.545 | 8051 | CCCCCC(=O)C |
| Adjuvant and guide herbs | caryophylli flos (ding xiang) | Methyl Salicylate | C8H8O3 | 152.16 | 42.55 | 0.652 | 4133 | COC(=O)C1=CC=CC=C1O |
| Adjuvant and guide herbs | caryophylli flos (ding xiang) | Moracin C | C19H18O4 | 310.37 | 82.13 | 0.29 | 155248 | CC(=CCC1=C(C=C(C=C1O)C2=CC3=C(O2)C=C(C=C3)O)O)C |
| Adjuvant and guide herbs | caryophylli flos (ding xiang) | Rhamnetin | C16H12O7 | 316.28 | 34 | 0.625 | 5281691 | COC1=CC(=C2C(=C1)OC(=C(C2=O)O)C3=CC(=C(C=C3)O)O)O |
| Adjuvant and guide herbs | caryophylli flos (ding xiang) | Strictosamide_qt | C26H30N2O8 | 498.53 | 76.3 | 0.76 | 10345799 | C=CC1C2CC3C4=C(CCN3C(=O)C2=COC1OC5C(C(C(C(O5)CO)O)O)O)C6=CC=CC=C6N4 |
| Adjuvant and guide herbs | caryophylli flos (ding xiang) | beta-sitosterol | C29H50O | 414.79 | 36.91 | 0.75 | 222284 | CCC(CCC(C)C1CCC2C1(CCC3C2CC=C4C3(CCC(C4)O)C)C)C(C)C |
| Adjuvant and guide herbs | caryophylli flos (ding xiang) | kaempferol | C15H10O6 | 286.25 | 41.88 | 0.24 | 5280863 | C1=CC(=CC=C1C2=C(C(=O)C3=C(C=C(C=C3O2)O)O)O)O |
| Adjuvant and guide herbs | caryophylli flos (ding xiang) | Stigmasterol | C29H48O | 412.77 | 43.83 | 0.76 | 5280794 | CCC(C=CC(C)C1CCC2C1(CCC3C2CC=C4C3(CCC(C4)O)C)C)C(C)C |
| Adjuvant and guide herbs | caryophylli flos (ding xiang) | quercetin | C15H10O7 | 302.25 | 46.43 | 0.28 | 5280343 | C1=CC(=C(C=C1C2=C(C(=O)C3=C(C=C(C=C3O2)O)O)O)O)O |
| Adjuvant and guide herbs | Borneolum or Borneolum Syntheticum(bing pian) | 7-Trimethylbicyclo[2.2.1]Heptan-2-Ol | C10H18O | 154.28 | 81.8 | 0.565 | 6552009 | CC1(C2CCC1(C(C2)O)C)C |
| Adjuvant and guide herbs | Borneolum or Borneolum Syntheticum(bing pian) | Caryophellene | C15H24 | 204.39 | 33.79 | 0.499 | 5498518 | CC1=CCCC(=C)C2CC(C2CC1)(C)C |
| Adjuvant and guide herbs | Borneolum or Borneolum Syntheticum(bing pian) | Borneol | C10H16O | 152.26 | 31.68 | 0.565 | 444294 | CC1(C2CCC1(C(=O)C2)C)C |
| Adjuvant and guide herbs | Borneolum or Borneolum Syntheticum(bing pian) | Camphor | C15H24 | 204.39 | 35.63 | 0.519 | 6918391 | CC(=C)C1CCC(C(C1)C(=C)C)(C)C=C |
| Adjuvant and guide herbs | Borneolum or Borneolum Syntheticum(bing pian) | Beta-Elemene | C15H24 | 204.39 | 35.63 | 0.58 | 6918391 | CC(=C)C1CCC(C(C1)C(=C)C)(C)C=C |
| Adjuvant and guide herbs | Borneolum or Borneolum Syntheticum(bing pian) | Isoborneol | C10H18O | 154.28 | 86.98 | 0.565 | 6321405 | CC1(C2CCC1(C(C2)O)C)C |
