# Supplementary material for: Guanxinshutong Alleviates Atherosclerosis by Suppressing Oxidative Stress and Proinflammation in ApoE−/− Mice
Source: Evid Based Complement Alternat Med. 2020 Sep 16;2020:1219371. doi: 10.1155/2020/1219371 (PMC7519182; doi:10.1155/2020/1219371)
Supplement: Supplementary Materials — Supplementary Table S1. Characteristics of active compounds in GXST. This file contains the herbs of GXST and herbs-associated compounds, molecular formula, molecular weight (MW), oral bioavailability (OB), drug-likeness, PubchemID, and SMILES. Supplementary Table S2. The potential targets of the compounds in GXST. This file contains the herbs' compounds and compound-associated targets, Uniprot ID and Gene Code. Supplementary Table S3. AS-related target. This file includes the gene name, target name, Uniprot ID, and the source of the database of AS-related target. Supplementary Table S4. Primer sequences for PCR. Supplementary Figure 1. The knock down efficiency of ApoE expression in mice. Total DNA was extracted from the heart of C57BL/6J wide type (liver) and ApoE−/− mice (liver, brain, heart, and aorta) according to the manufacturers's instructions. The DNA was used as a template to perform PCR with TaKaRa PCR Amplification Kit (TaKaRa Biotechnology) both in wide type (150 bp) and APOE deficiency mice (250 bp). M: DL 2000 marker; N: negative control; P: positive control; 1–8: ApoE−/− mice applied in our study; 9-10: C57BL/6J wide type (liver, liver). Supplementary Figure 2. The lower magnitude for HE, Masson staining (magnification: ×20), and CD68 of the aortic sinus (magnification: ×40). Supplementary Figure 3. The lower magnitude for IL-6, TNF-α and NF-κB of the aortic sinus (magnification: ×40). Supplementary Figure.4. GXST moderately improves LV remodeling. (A) Representative images of HE staining of left ventricular wall (n = 3). (B) Representative images of Masson staining of left ventricular wall (n = 3). (C) Quantitative analysis of the extracellular matrix in each group (n = 5). ∗P < 0.05 showed a significant difference compared with the Sham. #P < 0.05 showed a significant difference compared with the Model, ∗∗P < 0.01, ##P < 0.01. Supplementary Figure 5. The effects of GXST on the protein levels of inflammatory and oxidative stress factors in the aorta [file 1219371.f1.zip › 1219371.f1/Supplementary Table S2.docx]

Supplementary Table S2. The potential targets of the compounds in GXST

| **Compounds** | **Targets** | **Uniprot ID** | **Gene Code** |
| --- | --- | --- | --- |
| Glucose | KAT8 regulatory NSL complex subunit 3 | Q16706 | MAN2A1 |
| Glucose | Histo-blood group ABO system transferase | P16442 | ABO |
| Glucose | Beta-2 adrenergic receptor | P19961 | AMY2B |
| Glucose | Lactase-phlorizin hydrolase | P09848 | LCT |
| Glucose | Galectin-3 | P17931 | LGALS3 |
| Glucose | Glycolipid transfer protein | Q9NZD2 | GLTP |
| Glucose | Androgen receptor | O00244 | ATOX1 |
| Glucose | KAT8 regulatory NSL complex subunit 3 | P61626 | LYZ |
| Glucose | Actin, alpha skeletal muscle | P68133 | ACTA1 |
| Glucose | Androgen receptor | O15392 | C22 |
| Glucose | KAT8 regulatory NSL complex subunit 3 | P11226 | MBL2 |
| Glucose | Solute carrier family 22 member 8 | P11166 | SLC2A1 |
| Glucose | Sodium-dependent dopamine transporter | O60264 | SMARCA5 |
| Glucose | KAT8 regulatory NSL complex subunit 3 | O14594 | NCAN |
| Glucose | Interferon beta | P01574 | IFNB1 |
| Glucose | KAT8 regulatory NSL complex subunit 3 | P02144 | MB |
| Glucose | Beta-2 adrenergic receptor | P04745 | AMY1A |
| Glucose | Beta-2 adrenergic receptor | P04746 | AMY2A |
| Glucose | Galectin-2 | P05162 | LGALS2 |
| Glucose | Protein-tyrosine kinase 2-beta | P06737 | PYGL |
| Glucose | Protein-tyrosine kinase 2-beta | P11217 | PYGM |
| Glucose | Hexokinase-1 | P19367 | HK1 |
| Glucose | Serine/threonine-protein kinase pim-1 | P23219 | PTGS1 |
| Glucose | Corticosteroid-binding globulin | P35247 | SFTPD |
| Glucose | Mitogen-activated protein kinase kinase kinase kinase 2 | Q12851 | GCK |
| Glucose | Glucosamine-6-phosphate isomerase 1 | P46926 | GNPDA1 |
| Glucose | Galectin-7 | P47929 | LGALS7 |
| Glucose | Lactase-like protein | Q6UWM7 | LCTL |
| Glucose | Nuclear receptor subfamily 1 group I member 2 | Q9BW91 | NUDT9 |
| Glucose | Corticosteroid-binding globulin | Q9BZZ2 | SIGLEC1 |
| JujubosideB | Nuclear receptor subfamily 1 group I member 2 | P04150 | NR3C1 |
| JujubosideB | RuvB-like 2 | P08185 | SERPINA6 |
| JujubosideB | Neuronal acetylcholine receptor subunit alpha-7 | P08684 | CYP3A4 |
| JujubosideB | Neuronal acetylcholine receptor subunit alpha-7 | P10635 | CYP2D6 |
| JujubosideB | Carbonic anhydrase 2 | P23141 | CES1 |
| JujubosideB | Phospholipase A2 | P62258 | YWHAE |
| JujubosideB | Androgen receptor | P05023 | ATP1A1 |
| JujubosideB | Phospholipase A2 | P11473 | VDR |
| JujubosideB | Mitogen-activated protein kinase kinase kinase kinase 2 | O75311 | GLRA3 |
| JujubosideB | Bile salt export pump | P08183 | ABCB1 |
| JujubosideB | Baculoviral IAP repeat-containing protein 5 | P28472 | GABRB3 |
| JujubosideB | Multidrug resistance-associated protein 1 | P33527 | ABCC1 |
| JujubosideB | Canalicular multispecific organic anion transporter 1 | Q92887 | ABCC2 |
| JujubosideB | Broad substrate specificity ATP-binding cassette transporter ABCG2 | Q9UNQ0 | ABCG2 |
| JujubosideB | Beta-2 adrenergic receptor | Q8TES7 | ALB |
| JujubosideB | Neuronal acetylcholine receptor subunit alpha-7 | P05108 | CYP11A1 |
| JujubosideB | Serine/threonine-protein kinase pim-1 | P36873 | PPP1CC |
| JujubosideB | Solute carrier organic anion transporter family member 1A2 | P46721 | SLCO1A2 |
| JujubosideB | Sodium-dependent dopamine transporter | Q6ZQN7 | SLCO4C1 |
| JujubosideB | Sodium-dependent dopamine transporter | O94956 | SLCO2B1 |
| JujubosideB | Bile salt export pump | O95342 | ABCB11 |
| JujubosideB | Nuclear receptor subfamily 1 group I member 2 | P35372 | OSTalpha |
| JujubosideB | Nuclear receptor subfamily 1 group I member 2 | P35372 | OSTBETA |
| JujubosideB | Solute carrier family 22 member 8 | Q8TCC7 | SLC22A8 |
| JujubosideB | Sodium-dependent dopamine transporter | Q96BD0 | SLCO4A1 |
| JujubosideB | Sodium-dependent dopamine transporter | Q9NPD5 | SLCO1B3 |
| JujubosideB | Sodium-dependent dopamine transporter | Q9NYB5 | SLCO1C1 |
| JujubosideB | Sodium-dependent dopamine transporter | Q9Y6L6 | SLCO1B1 |
| JujubosideB | Neuronal acetylcholine receptor subunit alpha-7 | P04798 | CYP1A1 |
| JujubosideB | Androgen receptor | P13637 | ATP1A3 |
| JujubosideB | Androgen receptor | P50993 | ATP1A2 |
| [(2R)-5,7-dihydroxy-2-(4-hydroxyphenyl)chroman-4-one](http://lsp.nwu.edu.cn/molecule.php?qn=1040) | Serine/threonine-protein kinase pim-1 | P23219 | PTGS1 |
| (2R)-5,7-dihydroxy-2-(4-hydroxyphenyl)chroman-5-one | Estrogen receptor beta | Q92731 | ESR2 |
| (2R)-5,7-dihydroxy-2-(4-hydroxyphenyl)chroman-6-one | Serine/threonine-protein kinase pim-1 | P35354 | PTGS2 |
| (2R)-5,7-dihydroxy-2-(4-hydroxyphenyl)chroman-7-one | Hexokinase-1 | P08238 | HSP90AB1 |
| (2R)-5,7-dihydroxy-2-(4-hydroxyphenyl)chroman-8-one | Nuclear receptor subfamily 1 group I member 2 | P06401 | PGR |
| (2R)-5,7-dihydroxy-2-(4-hydroxyphenyl)chroman-9-one | Nuclear receptor subfamily 1 group I member 2 | P08235 | NR3C2 |
| [(-)-taxifolin](http://lsp.nwu.edu.cn/molecule.php?qn=1736) | Nuclear receptor subfamily 1 group I member 2 | P04150 | NR3C1 |
| (-)-taxifolin | Serine/threonine-protein kinase pim-1 | P23219 | PTGS1 |
| (-)-taxifolin | Hexokinase-1 | P08238 | HSP90AB1 |
| (-)-taxifolin | Phosphatidylinositol 4,5-bisphosphate 3-kinase catalytic subunit gamma isoform | P48736 | PIK3CG |
| beta-sitosterol | Nuclear receptor subfamily 1 group I member 2 | P06401 | PGR |
| beta-sitosterol | Nuclear receptor coactivator 2 | Q15596 | NCOA2 |
| beta-sitosterol | Serine/threonine-protein kinase pim-1 | P23219 | PTGS1 |
| beta-sitosterol | Hexokinase-1 | P08238 | HSP90AB1 |
| beta-sitosterol | Phosphatidylinositol 4,5-bisphosphate 3-kinase catalytic subunit gamma isoform | P48736 | PIK3CG |
| beta-sitosterol | Interferon beta | Q12809 | KCNH2 |
| beta-sitosterol | Serine/threonine-protein kinase pim-1 | P17612 | PRKACA |
| beta-sitosterol | D(1A) dopamine receptor | P21728 | DRD1 |
| beta-sitosterol | Muscarinic acetylcholine receptor M3 | P20309 | CHRM3 |
| beta-sitosterol | Muscarinic acetylcholine receptor M1 | P11229 | CHRM1 |
| beta-sitosterol | RuvB-like 2 | Q14524 | SCN5A |
| beta-sitosterol | Gamma-aminobutyric acid receptor subunit alpha-1 | P47869 | GABRA2 |
| beta-sitosterol | Muscarinic acetylcholine receptor M4 | P08173 | CHRM4 |
| beta-sitosterol | Nuclear receptor subfamily 1 group I member 2 | Q14432 | PDE3A |
| beta-sitosterol | Hexokinase-1 | P28223 | HTR2A |
| beta-sitosterol | Baculoviral IAP repeat-containing protein 5 | P31644 | GABRA5 |
| [beta-sitosterol](http://lsp.nwu.edu.cn/molecule.php?qn=1040) | Alpha-1A adrenergic receptor | P35348 | ADRA1A |
| [beta-sitosterol](http://lsp.nwu.edu.cn/molecule.php?qn=1040) | Gamma-aminobutyric acid receptor subunit alpha-3 | P34903 | GABRA3 |
| [beta-sitosterol](http://lsp.nwu.edu.cn/molecule.php?qn=1040) | Muscarinic acetylcholine receptor M2 | P08172 | CHRM2 |
| [beta-sitosterol](http://lsp.nwu.edu.cn/molecule.php?qn=1040) | Alpha-1B adrenergic receptor | P35368 | ADRA1B |
| [beta-sitosterol](http://lsp.nwu.edu.cn/molecule.php?qn=1040) | Beta-2 adrenergic receptor | P07550 | ADRB2 |
| beta-sitosterol | Muscarinic acetylcholine receptor M5 | Q15822 | CHRNA2 |
| [beta-sitosterol](http://lsp.nwu.edu.cn/molecule.php?qn=1736) | Solute carrier family 22 member 8 | P31645 | SLC6A4 |
| [beta-sitosterol](http://lsp.nwu.edu.cn/molecule.php?qn=1736) | Nuclear receptor subfamily 1 group I member 2 | P35372 | OPRM1 |
| [beta-sitosterol](http://lsp.nwu.edu.cn/molecule.php?qn=1736) | Gamma-aminobutyric acid receptor subunit alpha-1 | P14867 | GABRA1 |
| beta-sitosterol | Neuronal acetylcholine receptor subunit alpha-7 | P36544 | CHRNA7 |
| beta-sitosterol | Androgen receptor | P10415 | BCL2 |
| beta-sitosterol | Androgen receptor | Q07812 | BAX |
| beta-sitosterol | Carbonic anhydrase 2 | P55211 | CASP9 |
| beta-sitosterol | Interferon beta | P05412 | JUN |
| beta-sitosterol | Carbonic anhydrase 2 | P42574 | CASP3 |
| beta-sitosterol | Carbonic anhydrase 2 | Q14790 | CASP8 |
| beta-sitosterol | Serine/threonine-protein kinase pim-1 | Q9UGJ0 | PRKCA |
| beta-sitosterol | Signal transducer and activator of transcription 1-alpha/beta | P01137 | TGFB1 |
| beta-sitosterol | Serine/threonine-protein kinase pim-1 | P27169 | PON1 |
| beta-sitosterol | KAT8 regulatory NSL complex subunit 3 | P11137 | MAP2 |
| kaempferol | Nuclear receptor coactivator 2 | P60321 | NOS2 |
| kaempferol | Serine/threonine-protein kinase pim-1 | P23219 | PTGS1 |
| kaempferol | Androgen receptor | P10275 | AR |
| kaempferol | Serine/threonine-protein kinase pim-1 | P37231 | PPARG |
| kaempferol | Hexokinase-1 | P08238 | HSP90AB1 |
| kaempferol | Phosphatidylinositol 4,5-bisphosphate 3-kinase catalytic subunit gamma isoform | P48736 | PIK3CG |
| kaempferol | Serine/threonine-protein kinase pim-1 | P17612 | PRKACA |
| kaempferol | Nuclear receptor coactivator 2 | Q15596 | NCOA2 |
| kaempferol | Neuronal acetylcholine receptor subunit alpha-7 | P27487 | DPP4 |
| kaempferol | Serine/threonine-protein kinase pim-1 | P07477 | PRSS1 |
| kaempferol | Nuclear receptor subfamily 1 group I member 2 | P06401 | PGR |
| kaempferol | Estrogen receptor beta | P00734 | F2 |
| kaempferol | Muscarinic acetylcholine receptor M1 | P11229 | CHRM1 |
| kaempferol | Nuclear receptor coactivator 2 | P60323 | NOS3 |
| kaempferol | Gamma-aminobutyric acid receptor subunit alpha-1 | P14867 | GABRA1 |
| kaempferol | Acetylcholinesterase | P22303 | ACHE |
| kaempferol | Solute carrier family 22 member 8 | Q01959 | SLC6A3 |
| kaempferol | Muscarinic acetylcholine receptor M2 | P08172 | CHRM2 |
| kaempferol | Alpha-1B adrenergic receptor | P35368 | ADRA1B |
| kaempferol | DNA topoisomerase 2-beta | Q02880 | TOP2B |
| kaempferol | Estrogen receptor beta | P08709 | F7 |
| kaempferol | Carbonic anhydrase 2 | P0DP23 | CALM1 |
| kaempferol | Protein-tyrosine kinase 2-beta | Q04206 | RELA |
| kaempferol | Interferon beta | O14920 | IKBKB |
| kaempferol | Beta-2 adrenergic receptor | P31749 | AKT1 |
| kaempferol | Androgen receptor | P10415 | BCL2 |
| kaempferol | Androgen receptor | Q07812 | BAX |
| kaempferol | Tumor necrosis factor | P01375 | TNF |
| kaempferol | Interferon beta | P05412 | JUN |
| kaempferol | Beta-2 adrenergic receptor | O95433 | AHSA1 |
| kaempferol | Carbonic anhydrase 2 | P42574 | CASP3 |
| kaempferol | KAT8 regulatory NSL complex subunit 3 | P45983 | MAPK8 |
| kaempferol | Phospholipase A2 | P47989 | XDH |
| kaempferol | KAT8 regulatory NSL complex subunit 3 | P03956 | MMP1 |
| kaempferol | Signal transducer and activator of transcription 1-alpha/beta | P42224 | STAT1 |
| kaempferol | Carbonic anhydrase 2 | P06493 | CDC2 |
| kaempferol | Hexokinase-1 | P09601 | HMOX1 |
| kaempferol | Neuronal acetylcholine receptor subunit alpha-7 | P08684 | CYP3A4 |
| kaempferol | Neuronal acetylcholine receptor subunit alpha-7 | P05177 | CYP1A2 |
| kaempferol | Neuronal acetylcholine receptor subunit alpha-7 | P04798 | CYP1A1 |
| kaempferol | Hexokinase-1 | P05362 | ICAM1 |
| kaempferol | RuvB-like 2 | P16581 | SELE |
| kaempferol | Phospholipase A2 | P19320 | VCAM1 |
| kaempferol | Nuclear receptor subfamily 1 group I member 2 | O75469 | NR1I2 |
| kaempferol | Neuronal acetylcholine receptor subunit alpha-7 | Q16678 | CYP1B1 |
| kaempferol | Beta-2 adrenergic receptor | P09917 | ALOX5 |
| kaempferol | Glucosamine-6-phosphate isomerase 1 | Q92819 | HAS2 |
| kaempferol | Glucosamine-6-phosphate isomerase 1 | P09211 | GSTP1 |
| kaempferol | Beta-2 adrenergic receptor | P35869 | AHR |
| kaempferol | Serine/threonine-protein kinase pim-1 | O43242 | PSMD3 |
| kaempferol | Solute carrier family 22 member 8 | P14672 | SLC2A4 |
| kaempferol | Nuclear receptor subfamily 1 group I member 2 | Q14994 | NR1I3 |
| kaempferol | Interferon beta | P06213 | INSR |
| kaempferol | Neuronal acetylcholine receptor subunit alpha-7 | P49895 | DIO1 |
| kaempferol | Serine/threonine-protein kinase pim-1 | Q08209 | PPP3CA |
| kaempferol | Glucosamine-6-phosphate isomerase 1 | P09488 | GSTM1 |
| kaempferol | Glucosamine-6-phosphate isomerase 1 | P28161 | GSTM2 |
| kaempferol | Beta-2 adrenergic receptor | P42330 | AKR1C3 |
| naringenin | Serine/threonine-protein kinase pim-1 | P23219 | PTGS1 |
| naringenin | Estrogen receptor beta | Q92731 | ESR2 |
| naringenin | Hexokinase-1 | P08238 | HSP90AB1 |
| naringenin | Serine/threonine-protein kinase pim-1 | P17612 | PRKACA |
| naringenin | Phosphatidylinositol 4,5-bisphosphate 3-kinase catalytic subunit gamma isoform | P48736 | PIK3CG |
| naringenin | Protein-tyrosine kinase 2-beta | Q04206 | RELA |
| naringenin | Beta-2 adrenergic receptor | P31749 | AKT1 |
| naringenin | Androgen receptor | P10415 | BCL2 |
| naringenin | KAT8 regulatory NSL complex subunit 3 | P27361 | MAPK3 |
| naringenin | KAT8 regulatory NSL complex subunit 3 | P28482 | MAPK1 |
| naringenin | Carbonic anhydrase 2 | P42574 | CASP3 |
| naringenin | Estrogen receptor beta | P49327 | FASN |
| naringenin | Lactase-like protein | P01130 | LDLR |
| naringenin | Androgen receptor | Q92934 | BAD |
| naringenin | Sodium-dependent dopamine transporter | P00441 | SOD1 |
| naringenin | Carbonic anhydrase 2 | Q6IB77 | CAT |
| naringenin | KAT8 regulatory NSL complex subunit 3 | P55157 | MTTP |
| naringenin | Beta-2 adrenergic receptor | P04114 | APOB |
| naringenin | Serine/threonine-protein kinase pim-1 | Q6P1J6 | PLB1 |
| naringenin | Hexokinase-1 | P04035 | HMGCR |
| naringenin | Neuronal acetylcholine receptor subunit alpha-7 | P11511 | CYP19A1 |
| naringenin | Glucosamine-6-phosphate isomerase 1 | P09211 | GSTP1 |
| naringenin | Phospholipase A2 | P22309 | UGT1A1 |
| naringenin | Serine/threonine-protein kinase pim-1 | Q07869 | PPARA |
| naringenin | Sodium-dependent dopamine transporter | P36956 | SREBF1 |
| naringenin | Glucosamine-6-phosphate isomerase 1 | P00390 | GSR |
| naringenin | ATP-dependent translocase ABCB1 | P33527 | ABCC1 |
| naringenin | Adiponectin | Q15848 | ADIPOQ |
| naringenin | Sodium-dependent dopamine transporter | O75908 | SOAT2 |
| naringenin | Beta-2 adrenergic receptor | Q04828 | AKR1C1 |
| naringenin | Glucosamine-6-phosphate isomerase 1 | P17174 | GOT1 |
| naringenin | 4-aminobutyrate aminotransferase, mitochondrial | P80404 | ABAT |
| naringenin | Carbonic anhydrase 2 | P23141 | CES1 |
| naringenin | Sodium-dependent dopamine transporter | P35610 | SOAT1 |
| quercetin | Serine/threonine-protein kinase pim-1 | P23219 | PTGS1 |
| quercetin | Androgen receptor | P10275 | AR |
| quercetin | Hexokinase-1 | P08238 | HSP90AB1 |
| quercetin | Phosphatidylinositol 4,5-bisphosphate 3-kinase catalytic subunit gamma isoform | P48736 | PIK3CG |
| quercetin | Nuclear receptor coactivator 2 | Q15596 | NCOA2 |
| quercetin | Neuronal acetylcholine receptor subunit alpha-7 | P27487 | DPP4 |
| quercetin | Beta-2 adrenergic receptor | O95433 | AKR1A1ALDR1,ALR |
| quercetin | Serine/threonine-protein kinase pim-1 | P07477 | PRSS1 |
| quercetin | DNA topoisomerase 2-beta | Q02880 | TOP2B |
| quercetin | Interferon beta | Q12809 | KCNH2 |
| quercetin | RuvB-like 2 | Q14524 | SCN5A |
| quercetin | Estrogen receptor beta | Q9BQ89 | F10 |
| quercetin | Beta-2 adrenergic receptor | P07550 | ADRB2 |
| quercetin | KAT8 regulatory NSL complex subunit 3 | P08254 | MMP3 |
| quercetin | Serine/threonine-protein kinase pim-1 | P17612 | PRKACA |
| quercetin | RuvB-like 2 | P19793 | RXRA |
| quercetin | Acetylcholinesterase | P22303 | ACHE |
| quercetin | Gamma-aminobutyric acid receptor subunit alpha-1 | P14867 | GABRA1 |
| quercetin | KAT8 regulatory NSL complex subunit 3 | P27338 | MAOB |
| quercetin | Protein-tyrosine kinase 2-beta | Q04206 | **RELA** |
| quercetin | D(2) dopamine receptor | P00533 | EGFR |
| quercetin | Beta-2 adrenergic receptor | P31749 | AKT1 |
| quercetin | Phospholipase A2 | P15692 | VEGFA |
| quercetin | Carbonic anhydrase 2 | P24385 | CCND1 |
| quercetin | Androgen receptor | P10415 | BCL2 |
| quercetin | Androgen receptor | Q07817 | BCL2L1 |
| quercetin | Estrogen receptor beta | P01100 | FOS |
| quercetin | Carbonic anhydrase 2 | P38936 | CDKN1A |
| quercetin | D(2) dopamine receptor | P56537 | EIF6 |
| quercetin | Androgen receptor | Q07812 | BAX |
| quercetin | Carbonic anhydrase 2 | P55211 | CASP9 |
| quercetin | Serine/threonine-protein kinase pim-1 | P00749 | PLAU |
| quercetin | KAT8 regulatory NSL complex subunit 3 | P08253 | MMP2 |
| quercetin | KAT8 regulatory NSL complex subunit 3 | P14780 | MMP9 |
| quercetin | KAT8 regulatory NSL complex subunit 3 | P28482 | MAPK1 |
| quercetin | Interferon beta | P22301 | IL10 |
| quercetin | D(2) dopamine receptor | P01133 | EGF |
| quercetin | Interferon beta | P05412 | JUN |
| quercetin | Interferon beta | P05231 | IL6 |
| quercetin | Beta-2 adrenergic receptor | O95433 | AHSA1 |
| quercetin | Carbonic anhydrase 2 | P42574 | CASP3 |
| quercetin | Phospholipase A2 | P04637 | TP53 |
| quercetin | D(2) dopamine receptor | P19419 | ELK1 |
| quercetin | Nuclear receptor coactivator 2 | P25963 | NFKBIA |
| quercetin | Serine/threonine-protein kinase pim-1 | P16435 | POR |
| quercetin | Nuclear receptor subfamily 1 group I member 2 | P11926 | ODC1 |
| quercetin | Phospholipase A2 | P47989 | XDH |
| quercetin | Carbonic anhydrase 2 | Q14790 | CASP8 |
| quercetin | Tumor necrosis factor | P11387 | TOP1 |
| quercetin | Protein-tyrosine kinase 2-beta | P04049 | RAF1 |
| quercetin | Sodium-dependent dopamine transporter | P00441 | SOD1 |
| quercetin | Serine/threonine-protein kinase pim-1 | Q9UGJ0 | PRKCA |
| quercetin | KAT8 regulatory NSL complex subunit 3 | P03956 | MMP1 |
| quercetin | Glucosamine-6-phosphate isomerase 1 | Q16665 | HIF1A |
| quercetin | Signal transducer and activator of transcription 1-alpha/beta | P42224 | STAT1 |
| quercetin | Protein-tyrosine kinase 2-beta | Q06455 | RUNX1T1 |
| quercetin | Carbonic anhydrase 2 | P06493 | CDC2 |
| quercetin | Glucosamine-6-phosphate isomerase 1 | Q99527 | GRP78 |
| quercetin | D(2) dopamine receptor | P04626 | ERBB2 |
| quercetin | Acetyl-CoA carboxylase 1 | Q13085 | ACACA |
| quercetin | Hexokinase-1 | P09601 | HMOX1 |
| quercetin | Neuronal acetylcholine receptor subunit alpha-7 | P08684 | CYP3A4 |
| quercetin | Neuronal acetylcholine receptor subunit alpha-7 | P05177 | CYP1A2 |
| quercetin | Carbonic anhydrase 2 | Q03135 | CAV1 |
| quercetin | KAT8 regulatory NSL complex subunit 3 | P01106 | MYC |
| quercetin | Estrogen receptor beta | P13726 | F3 |
| quercetin | Mitogen-activated protein kinase kinase kinase kinase 2 | P17302 | GJA1 |
| quercetin | Neuronal acetylcholine receptor subunit alpha-7 | P04798 | CYP1A1 |
| quercetin | Interferon beta | P01584 | IL1B |
| quercetin | Carbonic anhydrase 2 | P13500 | CCL2 |
| quercetin | Phospholipase A2 | P19320 | VCAM1 |
| quercetin | Serine/threonine-protein kinase pim-1 | P43115 | PTGER3 |
| quercetin | Neuronal acetylcholine receptor subunit alpha-7 | P10145 | CXCL8 |
| quercetin | Serine/threonine-protein kinase pim-1 | P05771 | PRKCB |
| quercetin | Androgen receptor | O15392 | BIRC5 |
| quercetin | D(2) dopamine receptor | Q1HG44 | DUOXA2 |
| quercetin | Hexokinase-1 | Q00613 | HSF1 |
| quercetin | Signal transducer and activator of transcription 1-alpha/beta | P01137 | TGFB1 |
| quercetin | Signal transducer and activator of transcription 1-alpha/beta | P49888 | SULT1E1 |
| quercetin | KAT8 regulatory NSL complex subunit 3 | O43451 | MGAM |
| quercetin | Interferon beta | P60568 | IL2 |
| quercetin | Nuclear receptor subfamily 1 group I member 2 | O75469 | NR1I2 |
| quercetin | Neuronal acetylcholine receptor subunit alpha-7 | Q16678 | CYP1B1 |
| quercetin | Carbonic anhydrase 2 | P14635 | CCNB1 |
| quercetin | Serine/threonine-protein kinase pim-1 | P00750 | PLAT |
| quercetin | Signal transducer and activator of transcription 1-alpha/beta | P07204 | THBD |
| quercetin | RuvB-like 2 | P05121 | SERPINE1 |
| quercetin | Neuronal acetylcholine receptor subunit alpha-7 | P02452 | COL1A1 |
| quercetin | Interferon beta | P01579 | IFNG |
| quercetin | Interferon beta | P01583 | IL1A |
| quercetin | KAT8 regulatory NSL complex subunit 3 | P05164 | MPO |
| quercetin | Tumor necrosis factor | P11388 | TOP2A |
| quercetin | KAT8 regulatory NSL complex subunit 3 | P14598 | NCF1 |
| quercetin | Broad substrate specificity ATP-binding cassette transporter ABCG2 | Q9UNQ0 | ABCG2 |
| quercetin | Glucosamine-6-phosphate isomerase 1 | Q92819 | HAS2 |
| quercetin | Glucosamine-6-phosphate isomerase 1 | P09211 | GSTP1 |
| quercetin | Nuclear receptor coactivator 2 | Q16236 | NFE2L2 |
| quercetin | Nuclear receptor coactivator 2 | P15559 | NQO1 |
| quercetin | Nuclear receptor subfamily 1 group I member 2 | P09874 | PARP1 |
| quercetin | Beta-2 adrenergic receptor | P35869 | AHR |
| quercetin | Serine/threonine-protein kinase pim-1 | O43242 | PSMD3 |
| quercetin | Solute carrier family 22 member 8 | P14672 | SLC2A4 |
| quercetin | Neuronal acetylcholine receptor subunit alpha-7 | P02461 | COL3A1 |
| quercetin | Neuronal acetylcholine receptor subunit alpha-7 | O14625 | CXCL11 |
| quercetin | Neuronal acetylcholine receptor subunit alpha-7 | P19875 | CXCL2 |
| quercetin | Neuronal acetylcholine receptor subunit alpha-7 | Q96JK2 | DCAF5 |
| quercetin | Nuclear receptor subfamily 1 group I member 2 | Q14994 | NR1I3 |
| quercetin | Carbonic anhydrase 2 | O96017 | CHEK2 |
| quercetin | Interferon beta | P06213 | INSR |
| quercetin | Neuronal acetylcholine receptor subunit alpha-7 | O14493 | CLDN4 |
| quercetin | Serine/threonine-protein kinase pim-1 | Q03181 | PPARD |
| quercetin | Neuronal acetylcholine receptor subunit alpha-7 | P02741 | CRP |
| quercetin | Neuronal acetylcholine receptor subunit alpha-7 | P02778 | CXCL10 |
| quercetin | Neuronal acetylcholine receptor subunit alpha-7 | O15111 | CHUK |
| quercetin | Sodium-dependent dopamine transporter | Q9BX95 | SPP1 |
| quercetin | Protein-tyrosine kinase 2-beta | Q13950 | RUNX2 |
| quercetin | Protein-tyrosine kinase 2-beta | Q9NS23 | RASSF1 |
| quercetin | D(2) dopamine receptor | Q01094 | E2F1 |
| quercetin | Prostatic acid phosphatase | P15309 | ACPP |
| quercetin | Neuronal acetylcholine receptor subunit alpha-7 | P07339 | CTSD |
| quercetin | Interferon beta | P17936 | IGFBP3 |
| quercetin | Interferon beta | P01344 | IGF2 |
| quercetin | Carbonic anhydrase 2 | P29965 | CD40LG |
| quercetin | Interferon beta | P10914 | IRF1 |
| quercetin | D(2) dopamine receptor | P21860 | ERBB3 |
| quercetin | Neuronal acetylcholine receptor subunit alpha-7 | P49895 | DIO1 |
| quercetin | Nuclear receptor subfamily 1 group I member 2 | Q15113 | PCOLCE |
| quercetin | Nuclear receptor coactivator 2 | P55786 | NPEPPS |
| quercetin | Hexokinase-1 | P52789 | HK2 |
| quercetin | Nuclear receptor coactivator 2 | Q99801 | NKX3-1 |
| quercetin | Glucosamine-6-phosphate isomerase 1 | P09488 | GSTM1 |
| quercetin | Glucosamine-6-phosphate isomerase 1 | P28161 | GSTM2 |
| Salvilenone | Serine/threonine-protein kinase pim-1 | P23219 | PTGS1 |
| Salvilenone | Estrogen receptor beta | Q92731 | ESR2 |
| Salvilenone | Androgen receptor | P10275 | AR |
| Salvilenone | Muscarinic acetylcholine receptor M5 | P08912 | CHRM5 |
| Salvilenone | Serine/threonine-protein kinase pim-1 | P35354 | PTGS2 |
| Salvilenone | Hexokinase-1 | P46098 | HTR3A |
| Salvilenone | Serine/threonine-protein kinase pim-1 | P11309 | PIM1 |
| 3-beta-Hydroxymethyllenetanshiquinone | D(1A) dopamine receptor | P21728 | DRD1 |
| 3-beta-Hydroxymethyllenetanshiquinone | Estrogen receptor beta | P00734 | F2 |
| 3-beta-Hydroxymethyllenetanshiquinone | Muscarinic acetylcholine receptor M1 | P11229 | CHRM1 |
| 3-beta-Hydroxymethyllenetanshiquinone | Serine/threonine-protein kinase pim-1 | P35354 | PTGS2 |
| 3-beta-Hydroxymethyllenetanshiquinone | Carbonic anhydrase 2 | P00918 | CA2 |
| 3-beta-Hydroxymethyllenetanshiquinone | RuvB-like 2 | P19793 | RXRA |
| 3-beta-Hydroxymethyllenetanshiquinone | Nuclear receptor subfamily 1 group I member 2 | P41143 | OPRD1 |
| 3-beta-Hydroxymethyllenetanshiquinone | Acetylcholinesterase | P22303 | ACHE |
| 3-beta-Hydroxymethyllenetanshiquinone | Alpha-1A adrenergic receptor | P35348 | ADRA1A |
| 3-beta-Hydroxymethyllenetanshiquinone | Beta-2 adrenergic receptor | P07550 | ADRB2 |
| 3-beta-Hydroxymethyllenetanshiquinone | Nuclear receptor subfamily 1 group I member 2 | P35372 | OPRM1 |
| 3-beta-Hydroxymethyllenetanshiquinone | Neuronal acetylcholine receptor subunit alpha-7 | P27487 | DPP4 |
| 3-beta-Hydroxymethyllenetanshiquinone | Hexokinase-1 | P08238 | HSP90AB1 |
| 3-beta-Hydroxymethyllenetanshiquinone | Neuronal acetylcholine receptor subunit alpha-7 | P36544 | CHRNA7 |
| 3-beta-Hydroxymethyllenetanshiquinone | Interferon beta | P01857 | IGHG1 |
| 3-beta-Hydroxymethyllenetanshiquinone | Serine/threonine-protein kinase pim-1 | P07477 | PRSS1 |
| 5,6-dihydroxy-7-isopropyl-1,1-dimethyl-2,3-dihydrophenanthren-4-one | Serine/threonine-protein kinase pim-1 | P23219 | PTGS1 |
| 5,6-dihydroxy-7-isopropyl-1,1-dimethyl-2,3-dihydrophenanthren-4-one | Muscarinic acetylcholine receptor M3 | P20309 | CHRM3 |
| 5,6-dihydroxy-7-isopropyl-1,1-dimethyl-2,3-dihydrophenanthren-4-one | Muscarinic acetylcholine receptor M1 | P11229 | CHRM1 |
| 5,6-dihydroxy-7-isopropyl-1,1-dimethyl-2,3-dihydrophenanthren-4-one | RuvB-like 2 | Q14524 | SCN5A |
| 5,6-dihydroxy-7-isopropyl-1,1-dimethyl-2,3-dihydrophenanthren-4-one | Serine/threonine-protein kinase pim-1 | P35354 | PTGS2 |
| 5,6-dihydroxy-7-isopropyl-1,1-dimethyl-2,3-dihydrophenanthren-4-one | Carbonic anhydrase 2 | P00918 | CA2 |
| 5,6-dihydroxy-7-isopropyl-1,1-dimethyl-2,3-dihydrophenanthren-4-one | RuvB-like 2 | P19793 | RXRA |
| 5,6-dihydroxy-7-isopropyl-1,1-dimethyl-2,3-dihydrophenanthren-4-one | Acetylcholinesterase | P22303 | ACHE |
| 5,6-dihydroxy-7-isopropyl-1,1-dimethyl-2,3-dihydrophenanthren-4-one | Alpha-1A adrenergic receptor | P35348 | ADRA1A |
| 5,6-dihydroxy-7-isopropyl-1,1-dimethyl-2,3-dihydrophenanthren-4-one | Alpha-1B adrenergic receptor | P35368 | ADRA1B |
| 5,6-dihydroxy-7-isopropyl-1,1-dimethyl-2,3-dihydrophenanthren-4-one | Beta-2 adrenergic receptor | P07550 | ADRB2 |
| 5,6-dihydroxy-7-isopropyl-1,1-dimethyl-2,3-dihydrophenanthren-4-one | DNA topoisomerase 2-beta | Q02880 | TOP2B |
| 5,6-dihydroxy-7-isopropyl-1,1-dimethyl-2,3-dihydrophenanthren-4-one | Nuclear receptor subfamily 1 group I member 2 | P35372 | OPRM1 |
| 5,6-dihydroxy-7-isopropyl-1,1-dimethyl-2,3-dihydrophenanthren-4-one | Interferon beta | P01857 | IGHG1 |
| 5,6-dihydroxy-7-isopropyl-1,1-dimethyl-2,3-dihydrophenanthren-4-one | Nuclear receptor coactivator 2 | Q15596 | NCOA2 |
| 5,6-dihydroxy-7-isopropyl-1,1-dimethyl-2,3-dihydrophenanthren-4-one | Nuclear receptor coactivator 1 | Q15788 | NCOA1 |
| 5,6-dihydroxy-7-isopropyl-1,1-dimethyl-2,3-dihydrophenanthren-4-one | Carbonic anhydrase 2 | P0DP23 | CALM1 |
| 5,6-dihydroxy-7-isopropyl-1,1-dimethyl-2,3-dihydrophenanthren-4-one | D(1A) dopamine receptor | P21728 | DRD1 |
| 5,6-dihydroxy-7-isopropyl-1,1-dimethyl-2,3-dihydrophenanthren-4-one | Estrogen receptor beta | Q92731 | ESR2 |
| 5,6-dihydroxy-7-isopropyl-1,1-dimethyl-2,3-dihydrophenanthren-4-one | Androgen receptor | P10275 | AR |
| 5,6-dihydroxy-7-isopropyl-1,1-dimethyl-2,3-dihydrophenanthren-4-one | Serine/threonine-protein kinase pim-1 | P37231 | PPARG |
| 5,6-dihydroxy-7-isopropyl-1,1-dimethyl-2,3-dihydrophenanthren-4-one | Muscarinic acetylcholine receptor M5 | P08912 | CHRM5 |
| 5,6-dihydroxy-7-isopropyl-1,1-dimethyl-2,3-dihydrophenanthren-4-one | Nuclear receptor coactivator 2 | P60323 | NOS3 |
| 5,6-dihydroxy-7-isopropyl-1,1-dimethyl-2,3-dihydrophenanthren-4-one | Alpha-2A adrenergic receptor | P08913 | ADRA2A |
| 5,6-dihydroxy-7-isopropyl-1,1-dimethyl-2,3-dihydrophenanthren-4-one | Alpha-2C adrenergic receptor | P18825 | ADRA2C |
| 5,6-dihydroxy-7-isopropyl-1,1-dimethyl-2,3-dihydrophenanthren-4-one | Muscarinic acetylcholine receptor M4 | P08173 | CHRM4 |
| 5,6-dihydroxy-7-isopropyl-1,1-dimethyl-2,3-dihydrophenanthren-4-one | Nuclear receptor subfamily 1 group I member 2 | P41143 | OPRD1 |
| 5,6-dihydroxy-7-isopropyl-1,1-dimethyl-2,3-dihydrophenanthren-4-one | Hexokinase-1 | P28223 | HTR2A |
| 5,6-dihydroxy-7-isopropyl-1,1-dimethyl-2,3-dihydrophenanthren-4-one | Muscarinic acetylcholine receptor M2 | P08172 | CHRM2 |
| 5,6-dihydroxy-7-isopropyl-1,1-dimethyl-2,3-dihydrophenanthren-4-one | Solute carrier family 22 member 8 | Q01959 | SLC6A3 |
| 5,6-dihydroxy-7-isopropyl-1,1-dimethyl-2,3-dihydrophenanthren-4-one | Alpha-1D adrenergic receptor | P25100 | ADRA1D |
| 5,6-dihydroxy-7-isopropyl-1,1-dimethyl-2,3-dihydrophenanthren-4-one | Solute carrier family 22 member 8 | P31645 | SLC6A4 |
| 5,6-dihydroxy-7-isopropyl-1,1-dimethyl-2,3-dihydrophenanthren-4-one | D(2) dopamine receptor | P14416 | DRD2 |
| 5,6-dihydroxy-7-isopropyl-1,1-dimethyl-2,3-dihydrophenanthren-4-one | Gamma-aminobutyric acid receptor subunit alpha-1 | P14867 | GABRA1 |
| 5,6-dihydroxy-7-isopropyl-1,1-dimethyl-2,3-dihydrophenanthren-4-one | Neuronal acetylcholine receptor subunit alpha-7 | P36544 | CHRNA7 |
| 5,6-dihydroxy-7-isopropyl-1,1-dimethyl-2,3-dihydrophenanthren-4-one | Serine/threonine-protein kinase pim-1 | P17612 | PRKACA |
| 1-methyl-8,9-dihydro-7H-naphtho[5,6-g]benzofuran-6,10,11-trione | Serine/threonine-protein kinase pim-1 | P23219 | PTGS1 |
| 1-methyl-8,9-dihydro-7H-naphtho[5,6-g]benzofuran-6,10,11-trione | D(1A) dopamine receptor | P21728 | DRD1 |
| 1-methyl-8,9-dihydro-7H-naphtho[5,6-g]benzofuran-6,10,11-trione | Muscarinic acetylcholine receptor M3 | P20309 | CHRM3 |
| 1-methyl-8,9-dihydro-7H-naphtho[5,6-g]benzofuran-6,10,11-trione | Estrogen receptor beta | P00734 | F2 |
| 1-methyl-8,9-dihydro-7H-naphtho[5,6-g]benzofuran-6,10,11-trione | RuvB-like 2 | Q14524 | SCN5A |
| 1-methyl-8,9-dihydro-7H-naphtho[5,6-g]benzofuran-6,10,11-trione | Muscarinic acetylcholine receptor M5 | P08912 | CHRM5 |
| 1-methyl-8,9-dihydro-7H-naphtho[5,6-g]benzofuran-6,10,11-trione | Serine/threonine-protein kinase pim-1 | P35354 | PTGS2 |
| 1-methyl-8,9-dihydro-7H-naphtho[5,6-g]benzofuran-6,10,11-trione | Carbonic anhydrase 2 | P00918 | CA2 |
| 1-methyl-8,9-dihydro-7H-naphtho[5,6-g]benzofuran-6,10,11-trione | RuvB-like 2 | P19793 | RXRA |
| 1-methyl-8,9-dihydro-7H-naphtho[5,6-g]benzofuran-6,10,11-trione | Acetylcholinesterase | P22303 | ACHE |
| 1-methyl-8,9-dihydro-7H-naphtho[5,6-g]benzofuran-6,10,11-trione | Alpha-1A adrenergic receptor | P35348 | ADRA1A |
| 1-methyl-8,9-dihydro-7H-naphtho[5,6-g]benzofuran-6,10,11-trione | Beta-2 adrenergic receptor | P07550 | ADRB2 |
| 1-methyl-8,9-dihydro-7H-naphtho[5,6-g]benzofuran-6,10,11-trione | Nuclear receptor subfamily 1 group I member 2 | P35372 | OPRM1 |
| 1-methyl-8,9-dihydro-7H-naphtho[5,6-g]benzofuran-6,10,11-trione | Gamma-aminobutyric acid receptor subunit alpha-1 | P14867 | GABRA1 |
| 1-methyl-8,9-dihydro-7H-naphtho[5,6-g]benzofuran-6,10,11-trione | Neuronal acetylcholine receptor subunit alpha-7 | P27487 | DPP4 |
| 1-methyl-8,9-dihydro-7H-naphtho[5,6-g]benzofuran-6,10,11-trione | Hexokinase-1 | P08238 | HSP90AB1 |
| 1-methyl-8,9-dihydro-7H-naphtho[5,6-g]benzofuran-6,10,11-trione | Phosphatidylinositol 4,5-bisphosphate 3-kinase catalytic subunit gamma isoform | P48736 | PIK3CG |
| 1-methyl-8,9-dihydro-7H-naphtho[5,6-g]benzofuran-6,10,11-trione | Neuronal acetylcholine receptor subunit alpha-7 | P36544 | CHRNA7 |
| 1-methyl-8,9-dihydro-7H-naphtho[5,6-g]benzofuran-6,10,11-trione | Interferon beta | P01857 | IGHG1 |
| 1-methyl-8,9-dihydro-7H-naphtho[5,6-g]benzofuran-6,10,11-trione | Nuclear receptor coactivator 1 | Q15788 | NCOA1 |
| sugiol | Muscarinic acetylcholine receptor M3 | P20309 | CHRM3 |
| sugiol | Muscarinic acetylcholine receptor M1 | P11229 | CHRM1 |
| sugiol | RuvB-like 2 | Q14524 | SCN5A |
| sugiol | Muscarinic acetylcholine receptor M5 | P08912 | CHRM5 |
| sugiol | Serine/threonine-protein kinase pim-1 | P35354 | PTGS2 |
| sugiol | Carbonic anhydrase 2 | P00918 | CA2 |
| sugiol | Muscarinic acetylcholine receptor M4 | P08173 | CHRM4 |
| sugiol | Nuclear receptor subfamily 1 group I member 2 | P41143 | OPRD1 |
| sugiol | Acetylcholinesterase | P22303 | ACHE |
| sugiol | Alpha-1A adrenergic receptor | P35348 | ADRA1A |
| sugiol | Muscarinic acetylcholine receptor M2 | P08172 | CHRM2 |
| sugiol | Alpha-1B adrenergic receptor | P35368 | ADRA1B |
| sugiol | Beta-2 adrenergic receptor | P07550 | ADRB2 |
| sugiol | Alpha-1D adrenergic receptor | P25100 | ADRA1D |
| sugiol | D(2) dopamine receptor | P14416 | DRD2 |
| sugiol | Nuclear receptor subfamily 1 group I member 2 | P35372 | OPRM1 |
| luteolin | Serine/threonine-protein kinase pim-1 | P23219 | PTGS1 |
| luteolin | Androgen receptor | P10275 | AR |
| luteolin | Serine/threonine-protein kinase pim-1 | P35354 | PTGS2 |
| luteolin | Hexokinase-1 | P08238 | HSP90AB1 |
| luteolin | Serine/threonine-protein kinase pim-1 | P07477 | PRSS1 |
| luteolin | Nuclear receptor coactivator 2 | Q15596 | NCOA2 |
| luteolin | Serine/threonine-protein kinase pim-1 | P17612 | PRKACA |
| luteolin | Neuronal acetylcholine receptor subunit alpha-7 | P27487 | DPP4 |
| luteolin | Phosphatidylinositol 4,5-bisphosphate 3-kinase catalytic subunit gamma isoform | P48736 | PIK3CG |
| luteolin | Protein-tyrosine kinase 2-beta | Q04206 | RELA |
| luteolin | D(2) dopamine receptor | P00533 | EGFR |
| luteolin | Beta-2 adrenergic receptor | P31749 | AKT1 |
| luteolin | Phospholipase A2 | P15692 | VEGFA |
| luteolin | Carbonic anhydrase 2 | P24385 | CCND1 |
| luteolin | Androgen receptor | Q07817 | BCL2L1 |
| luteolin | Carbonic anhydrase 2 | P38936 | CDKN1A |
| luteolin | Carbonic anhydrase 2 | P55211 | CASP9 |
| luteolin | KAT8 regulatory NSL complex subunit 3 | P08253 | MMP2 |
| luteolin | KAT8 regulatory NSL complex subunit 3 | P14780 | MMP9 |
| luteolin | KAT8 regulatory NSL complex subunit 3 | P28482 | MAPK1 |
| luteolin | Interferon beta | P22301 | IL10 |
| luteolin | Carbonic anhydrase 2 | P11802 | CDK4 |
| luteolin | Tumor necrosis factor | P01375 | TNF |
| luteolin | Interferon beta | P05412 | JUN |
| luteolin | Interferon beta | P05231 | IL6 |
| luteolin | Carbonic anhydrase 2 | P42574 | CASP3 |
| luteolin | Phospholipase A2 | P04637 | TP53 |
| luteolin | Nuclear receptor coactivator 2 | P25963 | NFKBIA |
| luteolin | Phospholipase A2 | P47989 | XDH |
| luteolin | Tumor necrosis factor | P11387 | TOP1 |
| luteolin | KAT8 regulatory NSL complex subunit 3 | Q00987 | MDM2 |
| luteolin | Beta-2 adrenergic receptor | P05067 | APP |
| luteolin | KAT8 regulatory NSL complex subunit 3 | P03956 | MMP1 |
| luteolin | Nuclear receptor subfamily 1 group I member 2 | P12004 | PCNA |
| luteolin | D(2) dopamine receptor | P04626 | ERBB2 |
| luteolin | Serine/threonine-protein kinase pim-1 | P37231 | PPARG |
| luteolin | Hexokinase-1 | P09601 | HMOX1 |
| luteolin | Carbonic anhydrase 2 | P55210 | CASP7 |
| luteolin | Hexokinase-1 | P05362 | ICAM1 |
| luteolin | KAT8 regulatory NSL complex subunit 3 | Q07820 | MCL1 |
| luteolin | Androgen receptor | O15392 | BIRC5 |
| luteolin | Interferon beta | P60568 | IL2 |
| luteolin | Carbonic anhydrase 2 | P14635 | CCNB1 |
| luteolin | Phospholipase A2 | P14679 | TYR |
| luteolin | Interferon beta | P01579 | IFNG |
| luteolin | Interferon beta | P05112 | IL4 |
| luteolin | Tumor necrosis factor | P11388 | TOP2A |
| luteolin | Glucosamine-6-phosphate isomerase 1 | P09211 | GSTP1 |
| luteolin | Solute carrier family 22 member 8 | P14672 | SLC2A4 |
| luteolin | Interferon beta | P06213 | INSR |
| luteolin | Carbonic anhydrase 2 | P29965 | CD40LG |
| luteolin | Serine/threonine-protein kinase pim-1 | O14684 | PTGES |
| luteolin | Nuclear receptor subfamily 1 group I member 2 | Q9BZD4 | NUF2 |
| luteolin | Adenylate cyclase type 2 | Q08462 | ADCY2 |
| luteolin | KAT8 regulatory NSL complex subunit 3 | Q9NWH9 | MET |
| luteolin | Nuclear receptor subfamily 1 group I member 2 | P06401 | PGR |
| luteolin | D(1A) dopamine receptor | P21728 | DRD1 |
| luteolin | Muscarinic acetylcholine receptor M3 | P20309 | CHRM3 |
| luteolin | Estrogen receptor beta | P00734 | F2 |
| luteolin | Muscarinic acetylcholine receptor M1 | P11229 | CHRM1 |
| luteolin | RuvB-like 2 | Q14524 | SCN5A |
| luteolin | Muscarinic acetylcholine receptor M5 | P08912 | CHRM5 |
| luteolin | Carbonic anhydrase 2 | P00918 | CA2 |
| luteolin | RuvB-like 2 | P19793 | RXRA |
| luteolin | Nuclear receptor subfamily 1 group I member 2 | P41143 | OPRD1 |
| luteolin | Acetylcholinesterase | P22303 | ACHE |
| luteolin | Hexokinase-1 | P28223 | HTR2A |
| luteolin | Alpha-1A adrenergic receptor | P35348 | ADRA1A |
| luteolin | Muscarinic acetylcholine receptor M2 | P08172 | CHRM2 |
| luteolin | Beta-2 adrenergic receptor | P07550 | ADRB2 |
| luteolin | Solute carrier family 22 member 8 | P31645 | SLC6A4 |
| luteolin | Nuclear receptor subfamily 1 group I member 2 | P35372 | OPRM1 |
| luteolin | Gamma-aminobutyric acid receptor subunit alpha-1 | P14867 | GABRA1 |
| luteolin | Neuronal acetylcholine receptor subunit alpha-7 | P36544 | CHRNA7 |
| luteolin | Interferon beta | P01857 | IGHG1 |
| luteolin | Nuclear receptor coactivator 1 | Q15788 | NCOA1 |
| luteolin | Nuclear receptor coactivator 2 | P60321 | NOS2 |
| luteolin | Estrogen receptor beta | Q92731 | ESR2 |
| luteolin | Hexokinase-1 | P46098 | HTR3A |
| luteolin | Nuclear receptor subfamily 1 group I member 2 | Q14432 | PDE3A |
| luteolin | Alpha-1B adrenergic receptor | P35368 | ADRA1B |
| luteolin | Protein-tyrosine kinase 2-beta | P18031 | PTPN1 |
| luteolin | Solute carrier family 22 member 8 | Q01959 | SLC6A3 |
| luteolin | Alpha-1D adrenergic receptor | P25100 | ADRA1D |
| luteolin | Glucosamine-6-phosphate isomerase 1 | P49841 | GSK3B |
| luteolin | Serine/threonine-protein kinase pim-1 | P11309 | PIM1 |
| luteolin | Carbonic anhydrase 2 | P20248 | CCNA2 |
| 1,2,5,6-tetrahydrotanshinone | Serine/threonine-protein kinase pim-1 | P23219 | PTGS1 |
| 1,2,5,6-tetrahydrotanshinone | Muscarinic acetylcholine receptor M3 | P20309 | CHRM3 |
| 1,2,5,6-tetrahydrotanshinone | Muscarinic acetylcholine receptor M1 | P11229 | CHRM1 |
| 1,2,5,6-tetrahydrotanshinone | RuvB-like 2 | Q14524 | SCN5A |
| 1,2,5,6-tetrahydrotanshinone | Muscarinic acetylcholine receptor M5 | P08912 | CHRM5 |
| 1,2,5,6-tetrahydrotanshinone | Serine/threonine-protein kinase pim-1 | P35354 | PTGS2 |
| 1,2,5,6-tetrahydrotanshinone | Hexokinase-1 | P46098 | HTR3A |
| 1,2,5,6-tetrahydrotanshinone | Carbonic anhydrase 2 | P00918 | CA2 |
| 1,2,5,6-tetrahydrotanshinone | Muscarinic acetylcholine receptor M4 | P08173 | CHRM4 |
| 1,2,5,6-tetrahydrotanshinone | RuvB-like 2 | P19793 | RXRA |
| 1,2,5,6-tetrahydrotanshinone | Nuclear receptor subfamily 1 group I member 2 | P41143 | OPRD1 |
| 1,2,5,6-tetrahydrotanshinone | Nuclear receptor subfamily 1 group I member 2 | Q14432 | PDE3A |
| 1,2,5,6-tetrahydrotanshinone | Alpha-1A adrenergic receptor | P35348 | ADRA1A |
| 1,2,5,6-tetrahydrotanshinone | Muscarinic acetylcholine receptor M2 | P08172 | CHRM2 |
| 1,2,5,6-tetrahydrotanshinone | Alpha-1B adrenergic receptor | P35368 | ADRA1B |
| 1,2,5,6-tetrahydrotanshinone | Solute carrier family 22 member 8 | Q01959 | SLC6A3 |
| 1,2,5,6-tetrahydrotanshinone | Beta-2 adrenergic receptor | P07550 | ADRB2 |
| 1,2,5,6-tetrahydrotanshinone | Alpha-1D adrenergic receptor | P25100 | ADRA1D |
| 1,2,5,6-tetrahydrotanshinone | DNA topoisomerase 2-beta | Q02880 | TOP2B |
| 1,2,5,6-tetrahydrotanshinone | Nuclear receptor subfamily 1 group I member 2 | P35372 | OPRM1 |
| 1,2,5,6-tetrahydrotanshinone | Gamma-aminobutyric acid receptor subunit alpha-1 | P14867 | GABRA1 |
| 1,2,5,6-tetrahydrotanshinone | Hexokinase-1 | P08238 | HSP90AB1 |
| 1,2,5,6-tetrahydrotanshinone | Neuronal acetylcholine receptor subunit alpha-7 | P36544 | CHRNA7 |
| 1,2,5,6-tetrahydrotanshinone | Nuclear receptor coactivator 2 | Q15596 | NCOA2 |
| 1,2,5,6-tetrahydrotanshinone | Nuclear receptor coactivator 1 | Q15788 | NCOA1 |
| 1,2,5,6-tetrahydrotanshinone | D(1A) dopamine receptor | P21728 | DRD1 |
| 1,2,5,6-tetrahydrotanshinone | Hexokinase-1 | P28223 | HTR2A |
| 1,2,5,6-tetrahydrotanshinone | Solute carrier family 22 member 8 | P31645 | SLC6A4 |
| 1,2,5,6-tetrahydrotanshinone | Interferon beta | P01857 | IGHG1 |
| 1,2,5,6-tetrahydrotanshinone | Estrogen receptor beta | Q92731 | ESR2 |
| 1,2,5,6-tetrahydrotanshinone | Androgen receptor | P10275 | AR |
| 1,2,5,6-tetrahydrotanshinone | D(2) dopamine receptor | P14416 | DRD2 |
| 1,2,5,6-tetrahydrotanshinone | Nuclear receptor coactivator 2 | P60323 | NOS3 |
| 1,2,5,6-tetrahydrotanshinone | Alpha-2C adrenergic receptor | P18825 | ADRA2C |
| dan-shexinkumd | Nuclear receptor coactivator 2 | P60321 | NOS2 |
| dan-shexinkumd | Serine/threonine-protein kinase pim-1 | P23219 | PTGS1 |
| dan-shexinkumd | Estrogen receptor beta | P00734 | F2 |
| dan-shexinkumd | Interferon beta | Q12809 | KCNH2 |
| dan-shexinkumd | Muscarinic acetylcholine receptor M1 | P11229 | CHRM1 |
| dan-shexinkumd | Estrogen receptor beta | Q92731 | ESR2 |
| dan-shexinkumd | Androgen receptor | P10275 | AR |
| dan-shexinkumd | RuvB-like 2 | Q14524 | SCN5A |
| dan-shexinkumd | Serine/threonine-protein kinase pim-1 | P37231 | PPARG |
| dan-shexinkumd | Estrogen receptor beta | Q9BQ89 | F10 |
| dan-shexinkumd | Serine/threonine-protein kinase pim-1 | P35354 | PTGS2 |
| dan-shexinkumd | Carbonic anhydrase 2 | P00918 | CA2 |
| dan-shexinkumd | RuvB-like 2 | P19793 | RXRA |
| dan-shexinkumd | Acetylcholinesterase | P22303 | ACHE |
| dan-shexinkumd | Alpha-1B adrenergic receptor | P35368 | ADRA1B |
| dan-shexinkumd | Beta-2 adrenergic receptor | P07550 | ADRB2 |
| dan-shexinkumd | DNA topoisomerase 2-beta | Q02880 | TOP2B |
| dan-shexinkumd | Neuronal acetylcholine receptor subunit alpha-7 | P27487 | DPP4 |
| dan-shexinkumd | Glucosamine-6-phosphate isomerase 1 | P49841 | GSK3B |
| dan-shexinkumd | Carbonic anhydrase 2 | P24941 | CDK2 |
| dan-shexinkumd | Carbonic anhydrase 2 | O14757 | CHEK1 |
| dan-shexinkumd | Interferon beta | P01857 | IGHG1 |
| dan-shexinkumd | Serine/threonine-protein kinase pim-1 | P07477 | PRSS1 |
| dan-shexinkumd | Serine/threonine-protein kinase pim-1 | P11309 | PIM1 |
| dan-shexinkumd | Carbonic anhydrase 2 | P20248 | CCNA2 |
| dan-shexinkumd | Nuclear receptor coactivator 2 | Q15596 | NCOA2 |
| dan-shexinkumd | Nuclear receptor coactivator 1 | Q15788 | NCOA1 |
| dan-shexinkumd | Carbonic anhydrase 2 | P0DP23 | CALM1 |
| neocryptotanshinoneii | Serine/threonine-protein kinase pim-1 | P23219 | PTGS1 |
| neocryptotanshinoneii | D(1A) dopamine receptor | P21728 | DRD1 |
| neocryptotanshinoneii | Muscarinic acetylcholine receptor M3 | P20309 | CHRM3 |
| neocryptotanshinoneii | Muscarinic acetylcholine receptor M1 | P11229 | CHRM1 |
| neocryptotanshinoneii | Estrogen receptor beta | Q92731 | ESR2 |
| neocryptotanshinoneii | Androgen receptor | P10275 | AR |
| neocryptotanshinoneii | RuvB-like 2 | Q14524 | SCN5A |
| neocryptotanshinoneii | Serine/threonine-protein kinase pim-1 | P35354 | PTGS2 |
| neocryptotanshinoneii | Nuclear receptor coactivator 2 | P60323 | NOS3 |
| neocryptotanshinoneii | Carbonic anhydrase 2 | P00918 | CA2 |
| neocryptotanshinoneii | Muscarinic acetylcholine receptor M4 | P08173 | CHRM4 |
| neocryptotanshinoneii | RuvB-like 2 | P19793 | RXRA |
| neocryptotanshinoneii | Nuclear receptor subfamily 1 group I member 2 | P41143 | OPRD1 |
| neocryptotanshinoneii | Nuclear receptor subfamily 1 group I member 2 | Q14432 | PDE3A |
| neocryptotanshinoneii | Alpha-1A adrenergic receptor | P35348 | ADRA1A |
| neocryptotanshinoneii | Muscarinic acetylcholine receptor M2 | P08172 | CHRM2 |
| neocryptotanshinoneii | Alpha-1B adrenergic receptor | P35368 | ADRA1B |
| neocryptotanshinoneii | Solute carrier family 22 member 8 | Q01959 | SLC6A3 |
| neocryptotanshinoneii | Beta-2 adrenergic receptor | P07550 | ADRB2 |
| neocryptotanshinoneii | Alpha-1D adrenergic receptor | P25100 | ADRA1D |
| neocryptotanshinoneii | Solute carrier family 22 member 8 | P31645 | SLC6A4 |
| neocryptotanshinoneii | Nuclear receptor subfamily 1 group I member 2 | P35372 | OPRM1 |
| neocryptotanshinoneii | Gamma-aminobutyric acid receptor subunit alpha-1 | P14867 | GABRA1 |
| neocryptotanshinoneii | Glucosamine-6-phosphate isomerase 1 | P49841 | GSK3B |
| neocryptotanshinoneii | Hexokinase-1 | P08238 | HSP90AB1 |
| neocryptotanshinoneii | Carbonic anhydrase 2 | P24941 | CDK2 |
| neocryptotanshinoneii | Neuronal acetylcholine receptor subunit alpha-7 | P36544 | CHRNA7 |
| neocryptotanshinoneii | Serine/threonine-protein kinase pim-1 | P11309 | PIM1 |
| neocryptotanshinoneii | Carbonic anhydrase 2 | P20248 | CCNA2 |
| Vanillin | D(2) dopamine receptor | P03372 | ESR1 |
| Vanillin | Nuclear receptor subfamily 1 group I member 2 | P06401 | PGR |
| Vanillin | Androgen receptor | P10275 | AR |
| Vanillin | Estrogen receptor beta | Q92731 | ESR2 |
| Vanillin | Serine/threonine-protein kinase pim-1 | P04054 | PLA2G1B |
| Isoengelitin | Nuclear receptor subfamily 1 group I member 2 | O75469 | NR1I2 |
| Isoengelitin | Estrogen receptor beta | O95718 | ESRRB |
| Isoengelitin | Interferon beta | P01857 | IGHG1 |
| Isoengelitin | Phospholipase A2 | P02766 | TTR |
| Isoengelitin | D(2) dopamine receptor | P03372 | ESR1 |
| Isoengelitin | Corticosteroid-binding globulin | P04278 | SHBG |
| Isoengelitin | Neuronal acetylcholine receptor subunit alpha-7 | P05177 | CYP1A2 |
| Isoengelitin | ATP-dependent translocase ABCB1 | P08183 | ABCB1 |
| Isoengelitin | Tumor necrosis factor | P11388 | TOP2A |
| Isoengelitin | Estrogen receptor beta | P11474 | ESRRA |
| Isoengelitin | Beta-2 adrenergic receptor | P31749 | AKT1 |
| Isoengelitin | Multidrug resistance-associated protein 1 | P33527 | ABCC1 |
| Isoengelitin | Protein-tyrosine kinase 2-beta | Q14289 | PTK2B |
| Isoengelitin | Nuclear receptor coactivator 2 | Q15596 | NCOA2 |
| Isoengelitin | Nuclear receptor coactivator 1 | Q15788 | NCOA1 |
| Isoengelitin | Neuronal acetylcholine receptor subunit alpha-7 | Q16678 | CYP1B1 |
| Isoengelitin | Estrogen receptor beta | Q92731 | ESR2 |
| Isoengelitin | Glucosamine-6-phosphate isomerase 1 | Q99527 | GPER |
| Isoengelitin | ATP-binding cassette sub-family G member 2 | Q9UNQ0 | ABCG2 |
| Isoengelitin | Sodium-dependent dopamine transporter | O75908 | SOAT2 |
| Isoengelitin | Sodium-dependent dopamine transporter | P35610 | SOAT1 |
| Isoengelitin | KAT8 regulatory NSL complex subunit 3 | P55157 | MTTP |
| Isoengelitin | Neuronal acetylcholine receptor subunit alpha-7 | P08684 | CYP3A4 |
| Isoengelitin | Neuronal acetylcholine receptor subunit alpha-7 | P10632 | CYP2C8 |
| Isoengelitin | Neuronal acetylcholine receptor subunit alpha-7 | P10635 | CYP2D6 |
| Isoengelitin | Neuronal acetylcholine receptor subunit alpha-7 | P11712 | CYP2C9 |
| Isoengelitin | Carbonic anhydrase 2 | P16152 | CBR1 |
| Isoengelitin | Beta-2 adrenergic receptor | P42330 | AKR1C3 |
| Isoengelitin | Carbonic anhydrase 2 | Q00534 | CDK6 |
| Isoengelitin | Phospholipase A2 | Q6NUS8 | UGT3A1 |
| Isoengelitin | Androgen receptor | O43570 | CA12 |
| Isoengelitin | Androgen receptor | P00915 | CA1 |
| Isoengelitin | Carbonic anhydrase 2 | P00918 | CA2 |
| Isoengelitin | Neuronal acetylcholine receptor subunit alpha-7 | P04798 | CYP1A1 |
| Isoengelitin | Neuronal acetylcholine receptor subunit alpha-7 | P05181 | CYP2E1 |
| Isoengelitin | Serine/threonine-protein kinase pim-1 | P05771 | PRKCB |
| Isoengelitin | Carbonic anhydrase 2 | P07451 | CA3 |
| Isoengelitin | Serine/threonine-protein kinase pim-1 | Q9UGJ0 | PRKCA |
| Isoengelitin | Serine/threonine-protein kinase pim-1 | P17612 | PRKACA |
| Isoengelitin | Carbonic anhydrase 2 | P22748 | CA4 |
| Isoengelitin | Carbonic anhydrase 2 | P23280 | CA6 |
| Isoengelitin | Carbonic anhydrase 2 | P35218 | CA5A |
| Isoengelitin | Carbonic anhydrase 2 | P43166 | CA7 |
| Isoengelitin | Signal transducer and activator of transcription 1-alpha/beta | P43405 | SYK |
| Isoengelitin | Neuronal acetylcholine receptor subunit alpha-7 | P68400 | CSNK2A1 |
| Isoengelitin | Sodium-dependent dopamine transporter | Q14534 | SQLE |
| Isoengelitin | Carbonic anhydrase 2 | Q16790 | CA9 |
| Isoengelitin | Corticosteroid-binding globulin | Q4U2R8 | SLC22A6 |
| Isoengelitin | Corticosteroid-binding globulin | Q9NSA0 | SLC22A11 |
| Isoengelitin | Androgen receptor | Q9ULX7 | CA14 |
| Isoengelitin | Carbonic anhydrase 2 | Q9Y2D0 | CA5B |
| Isoengelitin | D(2) dopamine receptor | O00303 | EIF3F |
| Isoengelitin | Corticosteroid-binding globulin | O60669 | SLC16A7 |
| Isoengelitin | Signal transducer and activator of transcription 1-alpha/beta | O94768 | STK17B |
| Isoengelitin | Sodium-dependent dopamine transporter | O94956 | SLCO2B1 |
| Isoengelitin | Beta-2 adrenergic receptor | Q8TES7 | ALB |
| Isoengelitin | Androgen receptor | P06576 | ATP5B |
| Isoengelitin | Hexokinase-1 | P07900 | HSP90AA1 |
| Isoengelitin | Glucosamine-6-phosphate isomerase 1 | P08631 | HCK |
| Isoengelitin | Serine/threonine-protein kinase pim-1 | P11309 | PIM1 |
| Isoengelitin | Ribosyldihydronicotinamide dehydrogenase [quinone] | P16083 | NQO2 |
| Isoengelitin | Carbonic anhydrase 2 | P17676 | CEBPB |
| Isoengelitin | Neuronal acetylcholine receptor subunit alpha-7 | P21964 | COMT |
| Isoengelitin | Phospholipase A2 | P22314 | UBA1 |
| Isoengelitin | Interferon beta | P23458 | JAK1 |
| Isoengelitin | Androgen receptor | P25705 | ATP5A1 |
| Isoengelitin | Beta-2 adrenergic receptor | P35869 | AHR |
| Isoengelitin | Androgen receptor | P36542 | ATP5C1 |
| Isoengelitin | Phosphatidylinositol 4,5-bisphosphate 3-kinase catalytic subunit gamma isoform | P48736 | PIK3CG |
| Isoengelitin | Corticosteroid-binding globulin | P53985 | SLC16A1 |
| Isoengelitin | Hexokinase-1 | P54652 | HSPA2 |
| Isoengelitin | Actin, cytoplasmic 1 | P60709 | ACTB |
| Isoengelitin | Neuronal acetylcholine receptor subunit alpha-7 | P67870 | CSNK2B |
| Isoengelitin | Splicing factor 3B subunit 3 | Q15393 | SF3B3 |
| Isoengelitin | Glucosamine-6-phosphate isomerase 1 | Q6NVY1 | HIBCH |
| Isoengelitin | Canalicular multispecific organic anion transporter 1 | Q92887 | ABCC2 |
| Isoengelitin | RuvB-like 2 | Q9Y230 | RUVBL2 |
| Isoeugenitol | Interferon beta | P01857 | IGHG1 |
| Isoeugenitol | D(2) dopamine receptor | O00303 | EIF3F |
| Isoeugenitol | Corticosteroid-binding globulin | O60669 | SLC16A7 |
| Isoeugenitol | Nuclear receptor subfamily 1 group I member 2 | O75469 | NR1I2 |
| Isoeugenitol | Signal transducer and activator of transcription 1-alpha/beta | O94768 | STK17B |
| Isoeugenitol | Sodium-dependent dopamine transporter | O94956 | SLCO2B1 |
| Isoeugenitol | Beta-2 adrenergic receptor | Q8TES7 | ALB |
| Isoeugenitol | D(2) dopamine receptor | P03372 | ESR1 |
| Isoeugenitol | Corticosteroid-binding globulin | P04278 | SHBG |
| Isoeugenitol | Androgen receptor | P06576 | ATP5B |
| Isoeugenitol | Hexokinase-1 | P07900 | HSP90AA1 |
| Isoeugenitol | Multidrug resistance-associated protein 1 | P08183 | ABCB1 |
| Isoeugenitol | Glucosamine-6-phosphate isomerase 1 | P08631 | HCK |
| Isoeugenitol | Neuronal acetylcholine receptor subunit alpha-7 | P10632 | CYP2C8 |
| Isoeugenitol | Serine/threonine-protein kinase pim-1 | P11309 | PIM1 |
| Isoeugenitol | Ribosyldihydronicotinamide dehydrogenase [quinone] | P16083 | NQO2 |
| Isoeugenitol | Carbonic anhydrase 2 | P16152 | CBR1 |
| Isoeugenitol | Carbonic anhydrase 2 | P17676 | CEBPB |
| Isoeugenitol | Phospholipase A2 | P22314 | UBA1 |
| Isoeugenitol | Interferon beta | P23458 | JAK1 |
| Isoeugenitol | Androgen receptor | P25705 | ATP5A1 |
| Isoeugenitol | Multidrug resistance-associated protein 1 | P33527 | ABCC1 |
| Isoeugenitol | Beta-2 adrenergic receptor | P35869 | AHR |
| Isoeugenitol | Androgen receptor | P36542 | ATP5C1 |
| Isoeugenitol | Phosphatidylinositol 4,5-bisphosphate 3-kinase catalytic subunit gamma isoform | P48736 | PIK3CG |
| Isoeugenitol | Corticosteroid-binding globulin | P53985 | SLC16A1 |
| Isoeugenitol | Hexokinase-1 | P54652 | HSPA2 |
| Isoeugenitol | Actin, cytoplasmic 1 | P60709 | ACTB |
| Isoeugenitol | Neuronal acetylcholine receptor subunit alpha-7 | P67870 | CSNK2B |
| Isoeugenitol | Neuronal acetylcholine receptor subunit alpha-7 | P68400 | CSNK2A1 |
| Isoeugenitol | Splicing factor 3B subunit 3 | Q15393 | SF3B3 |
| Isoeugenitol | Neuronal acetylcholine receptor subunit alpha-7 | Q16678 | CYP1B1 |
| Isoeugenitol | Phospholipase A2 | Q6NUS8 | UGT3A1 |
| Isoeugenitol | Glucosamine-6-phosphate isomerase 1 | Q6NVY1 | HIBCH |
| Isoeugenitol | Estrogen receptor beta | Q92731 | ESR2 |
| Isoeugenitol | Canalicular multispecific organic anion transporter 1 | Q92887 | ABCC2 |
| Isoeugenitol | ATP-binding cassette sub-family G member 2 | Q9UNQ0 | ABCG2 |
| Isoeugenitol | RuvB-like 2 | Q9Y230 | RUVBL2 |
| Isoeugenitol | Estrogen receptor beta | O95718 | ESRRB |
| Isoeugenitol | Phospholipase A2 | P02766 | TTR |
| Isoeugenitol | Neuronal acetylcholine receptor subunit alpha-7 | P05177 | CYP1A2 |
| Isoeugenitol | Tumor necrosis factor | P11388 | TOP2A |
| Isoeugenitol | Estrogen receptor beta | P11474 | ESRRA |
| Isoeugenitol | Beta-2 adrenergic receptor | P31749 | AKT1 |
| Isoeugenitol | Protein-tyrosine kinase 2-beta | Q14289 | PTK2B |
| Isoeugenitol | Nuclear receptor coactivator 2 | Q15596 | NCOA2 |
| Isoeugenitol | Nuclear receptor coactivator 1 | Q15788 | NCOA1 |
| Isoeugenitol | Glucosamine-6-phosphate isomerase 1 | Q99527 | GPER |
| Isoeugenitol | Neuronal acetylcholine receptor subunit alpha-7 | P11511 | CYP19A1 |
| Isoeugenitol | Carbonic anhydrase 2 | Q00534 | CDK6 |
| Isoeugenitol | Beta-2 adrenergic receptor | Q04828 | AKR1C1 |
| Isoeugenitol | Interferon beta | Q9P2N6 | KIAA1310 |
| M-Methoxybenzaldehyde | Serine/threonine-protein kinase pim-1 | P04054 | PLA2G1B |
| Methyl-N-PentylKetone | Neuronal acetylcholine receptor subunit alpha-7 | P07108 | DBI |
| Methyl-N-PentylKetone | Protein-tyrosine kinase 2-beta | P08100 | RHO |
| Methyl-N-PentylKetone | Acyl-coenzyme A thioesterase 13 | Q9NPJ3 | ACOT13 |
| MethylSalicylate | Serine/threonine-protein kinase pim-1 | P04054 | PLA2G1B |
| MethylSalicylate | Interferon beta | O14920 | IKBKB |
| MethylSalicylate | Serine/threonine-protein kinase pim-1 | O43741 | PRKAB2 |
| MethylSalicylate | Beta-2 adrenergic receptor | Q8TES7 | ALB |
| MethylSalicylate | Phospholipase A2 | P04637 | TP53 |
| MethylSalicylate | ATP-binding cassette sub-family B member 1) | P08183 | ABCB1 |
| MethylSalicylate | Neuronal acetylcholine receptor subunit alpha-7 | P10632 | CYP2C8 |
| MethylSalicylate | Hexokinase-1 | P54652 | HSPA5 |
| MethylSalicylate | Neuronal acetylcholine receptor subunit alpha-7 | P11712 | CYP2C9 |
| MethylSalicylate | Nuclear receptor coactivator 2 | P19838 | NFKB1 |
| MethylSalicylate | Serine/threonine-protein kinase pim-1 | P23219 | PTGS1 |
| MethylSalicylate | D(2) dopamine receptor | P25101 | EDNRA |
| MethylSalicylate | Nuclear receptor coactivator 2 | P25963 | NFKBIA |
| MethylSalicylate | Neuronal acetylcholine receptor subunit alpha-7 | P33261 | CYP2C19 |
| MethylSalicylate | Serine/threonine-protein kinase pim-1 | P35354 | PTGS2 |
| MethylSalicylate | Protein-tyrosine kinase 2-beta | P51812 | RPS6KA3 |
| MethylSalicylate | Serine/threonine-protein kinase pim-1 | P54619 | PRKAG1 |
| MethylSalicylate | Serine/threonine-protein kinase pim-1 | P54646 | PRKAA2 |
| MethylSalicylate | Nuclear receptor coactivator 2 | Q00653 | NFKB2 |
| MethylSalicylate | Beta-2 adrenergic receptor | Q04828 | AKR1C1 |
| MethylSalicylate | Serine/threonine-protein kinase pim-1 | Q13131 | PRKAA1 |
| MethylSalicylate | Corticosteroid-binding globulin | Q4U2R8 | SLC22A6 |
| MethylSalicylate | Serine/threonine-protein kinase pim-1 | Q9UGJ0 | PRKAG2 |
| MethylSalicylate | Serine/threonine-protein kinase pim-1 | Q9Y478 | PRKAB1 |
| MethylSalicylate | Corticosteroid-binding globulin | Q9Y694 | SLC22A7 |
| MethylSalicylate | D(2) dopamine receptor | P03372 | ESR1 |
| MethylSalicylate | Nuclear receptor subfamily 1 group I member 2 | P06401 | PGR |
| MethylSalicylate | Androgen receptor | P10275 | AR |
| MethylSalicylate | Estrogen receptor beta | Q92731 | ESR2 |
| MoracinC | Serine/threonine-protein kinase pim-1 | P04054 | PLA2G1B |
| MoracinC | Interferon beta | O14920 | IKBKB |
| MoracinC | Serine/threonine-protein kinase pim-1 | O43741 | PRKAB2 |
| MoracinC | Beta-2 adrenergic receptor | Q8TES7 | ALB |
| MoracinC | Phospholipase A2 | P04637 | TP53 |
| MoracinC | ATP-binding cassette sub-family B member 1) | P08183 | ABCB1 |
| MoracinC | Neuronal acetylcholine receptor subunit alpha-7 | P10632 | CYP2C8 |
| MoracinC | Hexokinase-1 | P54652 | HSPA5 |
| MoracinC | Neuronal acetylcholine receptor subunit alpha-7 | P11712 | CYP2C9 |
| MoracinC | Nuclear receptor coactivator 2 | P19838 | NFKB1 |
| MoracinC | Serine/threonine-protein kinase pim-1 | P23219 | PTGS1 |
| MoracinC | D(2) dopamine receptor | P25101 | EDNRA |
| MoracinC | Nuclear receptor coactivator 2 | P25963 | NFKBIA |
| MoracinC | Neuronal acetylcholine receptor subunit alpha-7 | P33261 | CYP2C19 |
| MoracinC | Serine/threonine-protein kinase pim-1 | P35354 | PTGS2 |
| MoracinC | Protein-tyrosine kinase 2-beta | P51812 | RPS6KA3 |
| MoracinC | Serine/threonine-protein kinase pim-1 | P54619 | PRKAG1 |
| MoracinC | Serine/threonine-protein kinase pim-1 | P54646 | PRKAA2 |
| MoracinC | Nuclear receptor coactivator 2 | Q00653 | NFKB2 |
| MoracinC | Beta-2 adrenergic receptor | Q04828 | AKR1C1 |
| MoracinC | Serine/threonine-protein kinase pim-1 | Q13131 | PRKAA1 |
| MoracinC | Corticosteroid-binding globulin | Q4U2R8 | SLC22A6 |
| MoracinC | Serine/threonine-protein kinase pim-1 | Q9UGJ0 | PRKAG2 |
| MoracinC | Serine/threonine-protein kinase pim-1 | Q9Y478 | PRKAB1 |
| MoracinC | Corticosteroid-binding globulin | Q9Y694 | SLC22A7 |
| MoracinC | D(2) dopamine receptor | P03372 | ESR1 |
| MoracinC | Nuclear receptor subfamily 1 group I member 2 | P06401 | PGR |
| MoracinC | Androgen receptor | P10275 | AR |
| MoracinC | Estrogen receptor beta | Q92731 | ESR2 |
| Rhamnetin | ATP-binding cassette sub-family B member 1 | P08183 | ABCB1 |
| Rhamnetin | Tumor necrosis factor | P11388 | TOP2A |
| Rhamnetin | Estrogen receptor beta | P11474 | ESRRA |
| Rhamnetin | Beta-2 adrenergic receptor | P31749 | AKT1 |
| Rhamnetin | Multidrug resistance-associated protein 1 | P33527 | ABCC1 |
| Rhamnetin | Protein-tyrosine kinase 2-beta | Q14289 | PTK2B |
| Rhamnetin | Nuclear receptor coactivator 2 | Q15596 | NCOA2 |
| Rhamnetin | Nuclear receptor coactivator 1 | Q15788 | NCOA1 |
| Rhamnetin | Neuronal acetylcholine receptor subunit alpha-7 | Q16678 | CYP1B1 |
| Rhamnetin | Estrogen receptor beta | Q92731 | ESR2 |
| Rhamnetin | Glucosamine-6-phosphate isomerase 1 | Q99527 | GPER |
| Rhamnetin | ATP-binding cassette sub-family G member 2 | Q9UNQ0 | ABCG2 |
| Rhamnetin | Phospholipase A2 | Q6NUS8 | UGT3A1 |
| Rhamnetin | D(2) dopamine receptor | O00303 | EIF3F |
| Rhamnetin | Corticosteroid-binding globulin | O60669 | SLC16A7 |
| Rhamnetin | Nuclear receptor subfamily 1 group I member 2 | O75469 | NR1I2 |
| Rhamnetin | Signal transducer and activator of transcription 1-alpha/beta | O94768 | STK17B |
| Rhamnetin | Sodium-dependent dopamine transporter | O94956 | SLCO2B1 |
| Rhamnetin | Beta-2 adrenergic receptor | Q8TES7 | ALB |
| Rhamnetin | D(2) dopamine receptor | P03372 | ESR1 |
| Rhamnetin | Corticosteroid-binding globulin | P04278 | SHBG |
| Rhamnetin | Androgen receptor | P06576 | ATP5B |
| Rhamnetin | Hexokinase-1 | P07900 | HSP90AA1 |
| Rhamnetin | Glucosamine-6-phosphate isomerase 1 | P08631 | HCK |
| Rhamnetin | Neuronal acetylcholine receptor subunit alpha-7 | P10632 | CYP2C8 |
| Rhamnetin | Serine/threonine-protein kinase pim-1 | P11309 | PIM1 |
| Rhamnetin | Ribosyldihydronicotinamide dehydrogenase [quinone] | P16083 | NQO2 |
| Rhamnetin | Carbonic anhydrase 2 | P16152 | CBR1 |
| Rhamnetin | Carbonic anhydrase 2 | P17676 | CEBPB |
| Rhamnetin | Neuronal acetylcholine receptor subunit alpha-7 | P21964 | COMT |
| Rhamnetin | Phospholipase A2 | P22314 | UBA1 |
| Rhamnetin | Interferon beta | P23458 | JAK1 |
| Rhamnetin | Androgen receptor | P25705 | ATP5A1 |
| Rhamnetin | Beta-2 adrenergic receptor | P35869 | AHR |
| Rhamnetin | Androgen receptor | P36542 | ATP5C1 |
| Rhamnetin | Phosphatidylinositol 4,5-bisphosphate 3-kinase catalytic subunit gamma isoform | P48736 | PIK3CG |
| Rhamnetin | Corticosteroid-binding globulin | P53985 | SLC16A1 |
| Rhamnetin | Hexokinase-1 | P54652 | HSPA2 |
| Rhamnetin | Actin, cytoplasmic 1 | P60709 | ACTB |
| Rhamnetin | Neuronal acetylcholine receptor subunit alpha-7 | P67870 | CSNK2B |
| Rhamnetin | Neuronal acetylcholine receptor subunit alpha-7 | P68400 | CSNK2A1 |
| Rhamnetin | Splicing factor 3B subunit 3 | Q15393 | SF3B3 |
| Rhamnetin | Glucosamine-6-phosphate isomerase 1 | Q6NVY1 | HIBCH |
| Rhamnetin | Canalicular multispecific organic anion transporter 1 | Q92887 | ABCC2 |
| Rhamnetin | RuvB-like 2 | Q9Y230 | RUVBL2 |
| Rhamnetin | Sodium-dependent dopamine transporter | O75908 | SOAT2 |
| Rhamnetin | Neuronal acetylcholine receptor subunit alpha-7 | P05177 | CYP1A2 |
| Rhamnetin | Sodium-dependent dopamine transporter | P35610 | SOAT1 |
| Rhamnetin | KAT8 regulatory NSL complex subunit 3 | P55157 | MTTP |
| Rhamnetin | Carbonic anhydrase 2 | Q00534 | CDK6 |
| 7-Trimethylbicyclo[2.2.1]Heptan-2-Ol | Nuclear receptor subfamily 1 group I member 2 | Q14994 | NR1I3 |
| 7-Trimethylbicyclo[2.2.1]Heptan-2-Ol | Interferon beta | P01859 | IGHG2 |
| 7-Trimethylbicyclo[2.2.1]Heptan-2-Ol | D(2) dopamine receptor | P03372 | ESR1 |
| 7-Trimethylbicyclo[2.2.1]Heptan-2-Ol | Corticosteroid-binding globulin | P04278 | SHBG |
| 7-Trimethylbicyclo[2.2.1]Heptan-2-Ol | Neuronal acetylcholine receptor subunit alpha-7 | P05093 | CYP17A1 |
| 7-Trimethylbicyclo[2.2.1]Heptan-2-Ol | Neuronal acetylcholine receptor subunit alpha-7 | P05108 | CYP11A1 |
| 7-Trimethylbicyclo[2.2.1]Heptan-2-Ol | Nuclear receptor subfamily 1 group I member 2 | P08235 | NR3C2 |
| 7-Trimethylbicyclo[2.2.1]Heptan-2-Ol | Androgen receptor | P10275 | AR |
| 7-Trimethylbicyclo[2.2.1]Heptan-2-Ol | Neuronal acetylcholine receptor subunit alpha-7 | P11511 | CYP19A1 |
| 7-Trimethylbicyclo[2.2.1]Heptan-2-Ol | Hexokinase-1 | P14061 | HSD17B1 |
| 7-Trimethylbicyclo[2.2.1]Heptan-2-Ol | Beta-2 adrenergic receptor | P51857 | AKR1D1 |
| 7-Trimethylbicyclo[2.2.1]Heptan-2-Ol | Signal transducer and activator of transcription 1-alpha/beta | Q06520 | SULT2A1 |
| 7-Trimethylbicyclo[2.2.1]Heptan-2-Ol | Hexokinase-1 | Q8NBQ5 | HSD17B11 |
| 7-Trimethylbicyclo[2.2.1]Heptan-2-Ol | Estrogen receptor beta | Q92731 | ESR2 |
| 7-Trimethylbicyclo[2.2.1]Heptan-2-Ol | Phospholipase A2 | P11473 | VDR |
| 7-Trimethylbicyclo[2.2.1]Heptan-2-Ol | Gamma-aminobutyric acid receptor subunit alpha-1 | P14867 | GABRA1 |
| 7-Trimethylbicyclo[2.2.1]Heptan-2-Ol | Baculoviral IAP repeat-containing protein 5 | P31644 | GABRA5 |
| 7-Trimethylbicyclo[2.2.1]Heptan-2-Ol | Gamma-aminobutyric acid receptor subunit alpha-3 | P34903 | GABRA3 |
| 7-Trimethylbicyclo[2.2.1]Heptan-2-Ol | Gamma-aminobutyric acid receptor subunit alpha-1 | P47869 | GABRA2 |
| 7-Trimethylbicyclo[2.2.1]Heptan-2-Ol | Baculoviral IAP repeat-containing protein 5 | P48169 | GABRA4 |
| 7-Trimethylbicyclo[2.2.1]Heptan-2-Ol | Baculoviral IAP repeat-containing protein 5 | Q16445 | GABRA6 |
| 7-Trimethylbicyclo[2.2.1]Heptan-2-Ol | Phospholipase A2 | O75762 | TRPA1 |
| 7-Trimethylbicyclo[2.2.1]Heptan-2-Ol | Nuclear receptor subfamily 1 group I member 2 | P41145 | OPRK1 |
| 7-Trimethylbicyclo[2.2.1]Heptan-2-Ol | Phospholipase A2 | Q7Z2W7 | TRPM8 |
| 7-Trimethylbicyclo[2.2.1]Heptan-2-Ol | Phospholipase A2 | Q8NET8 | TRPV3 |
| 7-Trimethylbicyclo[2.2.1]Heptan-2-Ol | KAT8 regulatory NSL complex subunit 3 | P48449 | LSS |
| Borneol | Nuclear receptor subfamily 1 group I member 2 | Q14994 | NR1I3 |
| Borneol | Interferon beta | P01859 | IGHG2 |
| Borneol | D(2) dopamine receptor | P03372 | ESR1 |
| Borneol | Corticosteroid-binding globulin | P04278 | SHBG |
| Borneol | Neuronal acetylcholine receptor subunit alpha-7 | P05093 | CYP17A1 |
| Borneol | Neuronal acetylcholine receptor subunit alpha-7 | P05108 | CYP11A1 |
| Borneol | ATP-binding cassette sub-family B member 1 | P08183 | ABCB1 |
| Borneol | Nuclear receptor subfamily 1 group I member 2 | P08235 | NR3C2 |
| Borneol | Androgen receptor | P10275 | AR |
| Borneol | Neuronal acetylcholine receptor subunit alpha-7 | P11511 | CYP19A1 |
| Borneol | Hexokinase-1 | P14061 | HSD17B1 |
| Borneol | Beta-2 adrenergic receptor | P51857 | AKR1D1 |
| Borneol | Signal transducer and activator of transcription 1-alpha/beta | Q06520 | SULT2A1 |
| Borneol | Hexokinase-1 | Q8NBQ5 | HSD17B11 |
| Borneol | Estrogen receptor beta | Q92731 | ESR2 |
| Borneol | Phospholipase A2 | P11473 | VDR |
| Borneol | Gamma-aminobutyric acid receptor subunit alpha-1 | P14867 | GABRA1 |
| Borneol | Baculoviral IAP repeat-containing protein 5 | P31644 | GABRA5 |
| Borneol | Gamma-aminobutyric acid receptor subunit alpha-3 | P34903 | GABRA3 |
| Borneol | Gamma-aminobutyric acid receptor subunit alpha-1 | P47869 | GABRA2 |
| Borneol | Baculoviral IAP repeat-containing protein 5 | P48169 | GABRA4 |
| Borneol | Baculoviral IAP repeat-containing protein 5 | Q16445 | GABRA6 |
| Borneol | Phospholipase A2 | O75762 | TRPA1 |
| Borneol | Nuclear receptor subfamily 1 group I member 2 | P41145 | OPRK1 |
| Borneol | Phospholipase A2 | Q7Z2W7 | TRPM8 |
| Borneol | Phospholipase A2 | Q8NET8 | TRPV3 |
| Camphor | RuvB-like 2 | P19793 | RXRA |
| Camphor | Beta-2 adrenergic receptor | P51857 | AKR1D1 |
| Camphor | Nuclear receptor subfamily 1 group I member 2 | Q14994 | NR1I3 |
| Camphor | Nuclear receptor coactivator 1 | Q15788 | NCOA1 |
| Beta-Elemene | Neuronal acetylcholine receptor subunit alpha-7 | P29373 | CRABP2(0.85) |
| Isoborneol | Nuclear receptor subfamily 1 group I member 2 | Q14994 | NR1I3 |
| Isoborneol | Interferon beta | P01859 | IGHG2 |
| Isoborneol | D(2) dopamine receptor | P03372 | ESR1 |
| Isoborneol | Corticosteroid-binding globulin | P04278 | SHBG |
| Isoborneol | Neuronal acetylcholine receptor subunit alpha-7 | P05093 | CYP17A1 |
| Isoborneol | Neuronal acetylcholine receptor subunit alpha-7 | P05108 | CYP11A1 |
| Isoborneol | ATP-binding cassette sub-family B member 1 | P08183 | ABCB1 |
| Isoborneol | Nuclear receptor subfamily 1 group I member 2 | P08235 | NR3C2 |
| Isoborneol | Androgen receptor | P10275 | AR |
| Isoborneol | Neuronal acetylcholine receptor subunit alpha-7 | P11511 | CYP19A1 |
| Isoborneol | Hexokinase-1 | P14061 | HSD17B1 |
| Isoborneol | Beta-2 adrenergic receptor | P51857 | AKR1D1 |
| Isoborneol | Signal transducer and activator of transcription 1-alpha/beta | Q06520 | SULT2A1 |
| Isoborneol | Hexokinase-1 | Q8NBQ5 | HSD17B11 |
| Isoborneol | Estrogen receptor beta | Q92731 | ESR2 |
| Isoborneol | Phospholipase A2 | P11473 | VDR |
| Isoborneol | Gamma-aminobutyric acid receptor subunit alpha-1 | P14867 | GABRA1 |
| Isoborneol | Baculoviral IAP repeat-containing protein 5 | P31644 | GABRA5 |
| Isoborneol | Gamma-aminobutyric acid receptor subunit alpha-3 | P34903 | GABRA3 |
| Isoborneol | Gamma-aminobutyric acid receptor subunit alpha-1 | P47869 | GABRA2 |
| Isoborneol | Baculoviral IAP repeat-containing protein 5 | P48169 | GABRA4 |
| Isoborneol | Baculoviral IAP repeat-containing protein 5 | Q16445 | GABRA6 |
| Isoborneol | Phospholipase A2 | O75762 | TRPA1 |
| Isoborneol | Nuclear receptor subfamily 1 group I member 2 | P41145 | OPRK1 |
| Isoborneol | Phospholipase A2 | Q7Z2W7 | TRPM8 |
| Isoborneol | Phospholipase A2 | Q8NET8 | TRPV3 |
| Isoborneol | KAT8 regulatory NSL complex subunit 3 | P48449 | LSS |
