# Supplementary material for: Guanxinshutong Alleviates Atherosclerosis by Suppressing Oxidative Stress and Proinflammation in ApoE−/− Mice
Source: Evid Based Complement Alternat Med. 2020 Sep 16;2020:1219371. doi: 10.1155/2020/1219371 (PMC7519182; doi:10.1155/2020/1219371)
Supplement: Supplementary Materials — Supplementary Table S1. Characteristics of active compounds in GXST. This file contains the herbs of GXST and herbs-associated compounds, molecular formula, molecular weight (MW), oral bioavailability (OB), drug-likeness, PubchemID, and SMILES. Supplementary Table S2. The potential targets of the compounds in GXST. This file contains the herbs' compounds and compound-associated targets, Uniprot ID and Gene Code. Supplementary Table S3. AS-related target. This file includes the gene name, target name, Uniprot ID, and the source of the database of AS-related target. Supplementary Table S4. Primer sequences for PCR. Supplementary Figure 1. The knock down efficiency of ApoE expression in mice. Total DNA was extracted from the heart of C57BL/6J wide type (liver) and ApoE−/− mice (liver, brain, heart, and aorta) according to the manufacturers's instructions. The DNA was used as a template to perform PCR with TaKaRa PCR Amplification Kit (TaKaRa Biotechnology) both in wide type (150 bp) and APOE deficiency mice (250 bp). M: DL 2000 marker; N: negative control; P: positive control; 1–8: ApoE−/− mice applied in our study; 9-10: C57BL/6J wide type (liver, liver). Supplementary Figure 2. The lower magnitude for HE, Masson staining (magnification: ×20), and CD68 of the aortic sinus (magnification: ×40). Supplementary Figure 3. The lower magnitude for IL-6, TNF-α and NF-κB of the aortic sinus (magnification: ×40). Supplementary Figure.4. GXST moderately improves LV remodeling. (A) Representative images of HE staining of left ventricular wall (n = 3). (B) Representative images of Masson staining of left ventricular wall (n = 3). (C) Quantitative analysis of the extracellular matrix in each group (n = 5). ∗P < 0.05 showed a significant difference compared with the Sham. #P < 0.05 showed a significant difference compared with the Model, ∗∗P < 0.01, ##P < 0.01. Supplementary Figure 5. The effects of GXST on the protein levels of inflammatory and oxidative stress factors in the aorta [file 1219371.f1.zip › 1219371.f1/Supplementary Table S3.docx]

Supplementary Table S3. AS-related target

| **Gene name** | **Target name** | **UniProt ID** | **Database** |
| --- | --- | --- | --- |
| ABCA1 | ATP Binding Cassette Subfamily A Member 1 | O95477 | GeneCards |
| ABCC6 | ATP Binding Cassette Subfamily C Member 6 | O95255 | GeneCards |
| ABCG1 | ATP Binding Cassette Subfamily G Member 1 | P45844 | GeneCards |
| ABCG5 | ATP Binding Cassette Subfamily G Member 5 | Q9H222 | GeneCards |
| ABCG8 | ATP Binding Cassette Subfamily G Member 8 | Q9H221 | GeneCards |
| ACE | Angiotensin I Converting Enzyme | P12821 | GeneCards |
| ACTA2 | Actin Alpha 2, Smooth Muscle | P62736 | GeneCards |
| ADIPOQ | Adiponectin, C1Q And Collagen Domain Containing | Q15848 | GeneCards |
| AGER | Advanced Glycosylation End-Product Specific Receptor | Q15109 | GeneCards |
| AGT | Angiotensinogen | P01019 | GeneCards |
| AGTR1 | Angiotensin II Receptor Type 1 | P30556 | GeneCards |
| ALB | Albumin | P02768 | GeneCards |
| ALOX5 | Arachidonate 5-Lipoxygenase | P09917 | GeneCards |
| ANGPTL3 | Angiopoietin Like 3 | Q9Y5C1 | GeneCards |
| APOA1 | Apolipoprotein A1 | P02647 | GeneCards |
| APOA2 | Apolipoprotein A2 | P02652 | GeneCards |
| APOA5 | Apolipoprotein A5 | Q6Q788 | GeneCards |
| APOB | Apolipoprotein B | P04114 | GeneCards |
| APOC2 | Apolipoprotein C2 | P02655 | GeneCards |
| APOC3 | Apolipoprotein C3 | P02656 | GeneCards |
| APOE | Apolipoprotein E | P02649 | GeneCards |
| ATHS | Atherosclerosis Susceptibility (Lipoprotein Associated) | O75531 | GeneCards |
| BANF1 | Barrier To Autointegration Factor 1 | P04040 | GeneCards |
| CAT | Catalase | Q6IB77 | GeneCards |
| CCL2 | C-C Motif Chemokine Ligand 2 | P13500 | GeneCards |
| CDKN2B-AS1 | CDKN2B Antisense RNA 1 | Q9UM00 | GeneCards |
| CETP | Cholesteryl Ester Transfer Protein | P11597 | GeneCards |
| COG2 | Component Of Oligomeric Golgi Complex 2 | Q14746 | GeneCards |
| COL3A1 | Collagen Type III Alpha 1 Chain | P02461 | GeneCards |
| CRP | C-Reactive Protein | P02741 | GeneCards |
| CX3CR1 | C-X3-C Motif Chemokine Receptor 1 | P49238 | GeneCards |
| CYBA | cytochrome b-245 alpha chain | P13498 | DisGeNET |
| CYP27A1 | Cytochrome P450 Family 27 Subfamily A Member 1 | Q02318 | GeneCards |
| CYP7A1 | Cytochrome P450 Family 7 Subfamily A Member 1 | P22680 | GeneCards |
| EDN1 | Endothelin 1 | P05305 | GeneCards |
| ELN | Elastin | P15502 | GeneCards |
| ENG | Endoglin | P17813 | GeneCards |
| ENPP1 | Ectonucleotide Pyrophosphatase/Phosphodiesterase 1 | P22413 | GeneCards |
| EPHX2 | Epoxide Hydrolase 2 | P34913 | GeneCards |
| ESR1 | Estrogen Receptor 1 | P03372 | GeneCards |
| F2 | Coagulation Factor II, Thrombin | P00734 | GeneCards |
| F3 | Coagulation Factor III, Tissue Factor | P13726 | GeneCards |
| F5 | Coagulation Factor V | P12259 | GeneCards |
| F7 | Coagulation Factor VII | P08709 | GeneCards |
| FBN1 | Fibrillin 1 | P35555 | GeneCards |
| GGCX | Gamma-Glutamyl Carboxylase | P38435 | GeneCards |
| HMGCR | 3-Hydroxy-3-Methylglutaryl-CoA Reductase | P04035 | GeneCards |
| HP | haptoglobin | P00738 | DisGeNET |
| ICAM1 | intercellular adhesion molecule 1 | P05362 | DisGeNET |
| IFNG | interferon gamma | P01579 | DisGeNET |
| IL10 | Interleukin 10 | P22301 | GeneCards |
| IL1B | Interleukin 1 Beta | P01584 | GeneCards |
| IL6 | interleukin 6 | P05231 | DisGeNET |
| INS | Insulin | P01308 | GeneCards |
| ITGB3 | Integrin Subunit Beta 3 | P05106 | GeneCards |
| LCAT | Lecithin-Cholesterol Acyltransferase | P04180 | GeneCards |
| LDLR | Low Density Lipoprotein Receptor | P01130 | GeneCards |
| LDLRAP1 | Low Density Lipoprotein Receptor Adaptor Protein 1 | Q5SW96 | GeneCards |
| LEP | Leptin | P41159 | GeneCards |
| LIPA | Lipase A, Lysosomal Acid Type | P38571 | GeneCards |
| LIPC | Lipase C, Hepatic Type | P11150 | GeneCards |
| LMNA | Lamin A/C | P02545 | GeneCards |
| LOX | Lysyl Oxidase | P28300 | GeneCards |
| LPA | Lipoprotein(A) | P08519 | GeneCards |
| LPL | Lipoprotein Lipase | P06858 | GeneCards |
| MAPT | Microtubule Associated Protein Tau | P10636 | GeneCards |
| MMP2 | Matrix Metallopeptidase 2 | P08253 | GeneCards |
| MMP3 | Matrix Metallopeptidase 3 | P08254 | GeneCards |
| MMP9 | Matrix Metallopeptidase 9 | P14780 | GeneCards |
| MPO | Myeloperoxidase | P05164 | GeneCards |
| MTHFR | Methylenetetrahydrofolate Reductase | P42898 | GeneCards |
| MTTP | Microsomal Triglyceride Transfer Protein | P55157 | GeneCards |
| MYH11 | Myosin Heavy Chain 11 | P35749 | GeneCards |
| NOS3 | Nitric Oxide Synthase 3 | P29474 | GeneCards |
| NOTCH3 | Notch Receptor 3 | Q9UM47 | GeneCards |
| OLR1 | Oxidized Low Density Lipoprotein Receptor 1 | P78380 | GeneCards |
| PCSK9 | Proprotein Convertase Subtilisin/Kexin Type 9 | Q8NBP7 | GeneCards |
| PECAM1 | Platelet And Endothelial Cell Adhesion Molecule 1 | P16284 | GeneCards |
| PLA2G2A | phospholipase A2 group IIA | P14555 | DisGeNET |
| PLA2G7 | Phospholipase A2 Group VII | Q13093 | GeneCards |
| PLAT | Plasminogen Activator, Tissue Type | P00750 | GeneCards |
| PLTP | Phospholipid Transfer Protein | P55058 | GeneCards |
| PON1 | paraoxonase 1 | P27169 | DisGeNET |
| PON2 | Paraoxonase 2 | Q15165 | GeneCards |
| PPARA | Peroxisome Proliferator Activated Receptor Alpha | Q07869 | GeneCards |
| PPARG | peroxisome proliferator activated receptor gamma | P37231 | DisGeNET |
| PTGIS | Prostaglandin I2 Synthase | Q16647 | GeneCards |
| PTGS2 | prostaglandin-endoperoxide synthase 2 | P35354 | DisGeNET |
| RETN | Resistin | Q9HD89 | GeneCards |
| SCARB1 | Scavenger Receptor Class B Member 1 | Q8WTV0 | GeneCards |
| SELE | Selectin E | P16581 | GeneCards |
| SELP | Selectin P | P16109 | GeneCards |
| SERPINC1 | Serpin Family C Member 1 | P01008 | GeneCards |
| SERPINE1 | serpin family E member 1 | P05121 | DisGeNET |
| SIRT1 | sirtuin 1 | Q96EB6 | DisGeNET |
| SOAT1 | Sterol O-Acyltransferase 1 | P35610 | GeneCards |
| SPP1 | Secreted Phosphoprotein 1 | Q9BX95 | MalaCards |
| THBD | Thrombomodulin | P07204 | GeneCards |
| TLR4 | Toll Like Receptor 4 | O00206 | GeneCards |
| TNF | Tumor Necrosis Factor | P01375 | GeneCards |
| TNFRSF11B | TNF Receptor Superfamily Member 11b | O00300 | GeneCards |
| VCAM1 | Vascular Cell Adhesion Molecule 1 | P19320 | GeneCards |
| VEGFA | Vascular Endothelial Growth Factor A | P15692 | GeneCards |
| VWF | Von Willebrand Factor | P04275 | GeneCards |
| WRN | WRN RecQ Like Helicase | Q14191 | GeneCards |
| ZMPSTE24 | Zinc Metallopeptidase STE24 | O75844 | GeneCards |
