# Supplementary material for: Guanxinshutong Alleviates Atherosclerosis by Suppressing Oxidative Stress and Proinflammation in ApoE−/− Mice
Source: Evid Based Complement Alternat Med. 2020 Sep 16;2020:1219371. doi: 10.1155/2020/1219371 (PMC7519182; doi:10.1155/2020/1219371)
Supplement: Supplementary Materials — Supplementary Table S1. Characteristics of active compounds in GXST. This file contains the herbs of GXST and herbs-associated compounds, molecular formula, molecular weight (MW), oral bioavailability (OB), drug-likeness, PubchemID, and SMILES. Supplementary Table S2. The potential targets of the compounds in GXST. This file contains the herbs' compounds and compound-associated targets, Uniprot ID and Gene Code. Supplementary Table S3. AS-related target. This file includes the gene name, target name, Uniprot ID, and the source of the database of AS-related target. Supplementary Table S4. Primer sequences for PCR. Supplementary Figure 1. The knock down efficiency of ApoE expression in mice. Total DNA was extracted from the heart of C57BL/6J wide type (liver) and ApoE−/− mice (liver, brain, heart, and aorta) according to the manufacturers's instructions. The DNA was used as a template to perform PCR with TaKaRa PCR Amplification Kit (TaKaRa Biotechnology) both in wide type (150 bp) and APOE deficiency mice (250 bp). M: DL 2000 marker; N: negative control; P: positive control; 1–8: ApoE−/− mice applied in our study; 9-10: C57BL/6J wide type (liver, liver). Supplementary Figure 2. The lower magnitude for HE, Masson staining (magnification: ×20), and CD68 of the aortic sinus (magnification: ×40). Supplementary Figure 3. The lower magnitude for IL-6, TNF-α and NF-κB of the aortic sinus (magnification: ×40). Supplementary Figure.4. GXST moderately improves LV remodeling. (A) Representative images of HE staining of left ventricular wall (n = 3). (B) Representative images of Masson staining of left ventricular wall (n = 3). (C) Quantitative analysis of the extracellular matrix in each group (n = 5). ∗P < 0.05 showed a significant difference compared with the Sham. #P < 0.05 showed a significant difference compared with the Model, ∗∗P < 0.01, ##P < 0.01. Supplementary Figure 5. The effects of GXST on the protein levels of inflammatory and oxidative stress factors in the aorta [file 1219371.f1.zip › 1219371.f1/supplementaty figures files.pptx]

## Slide 1
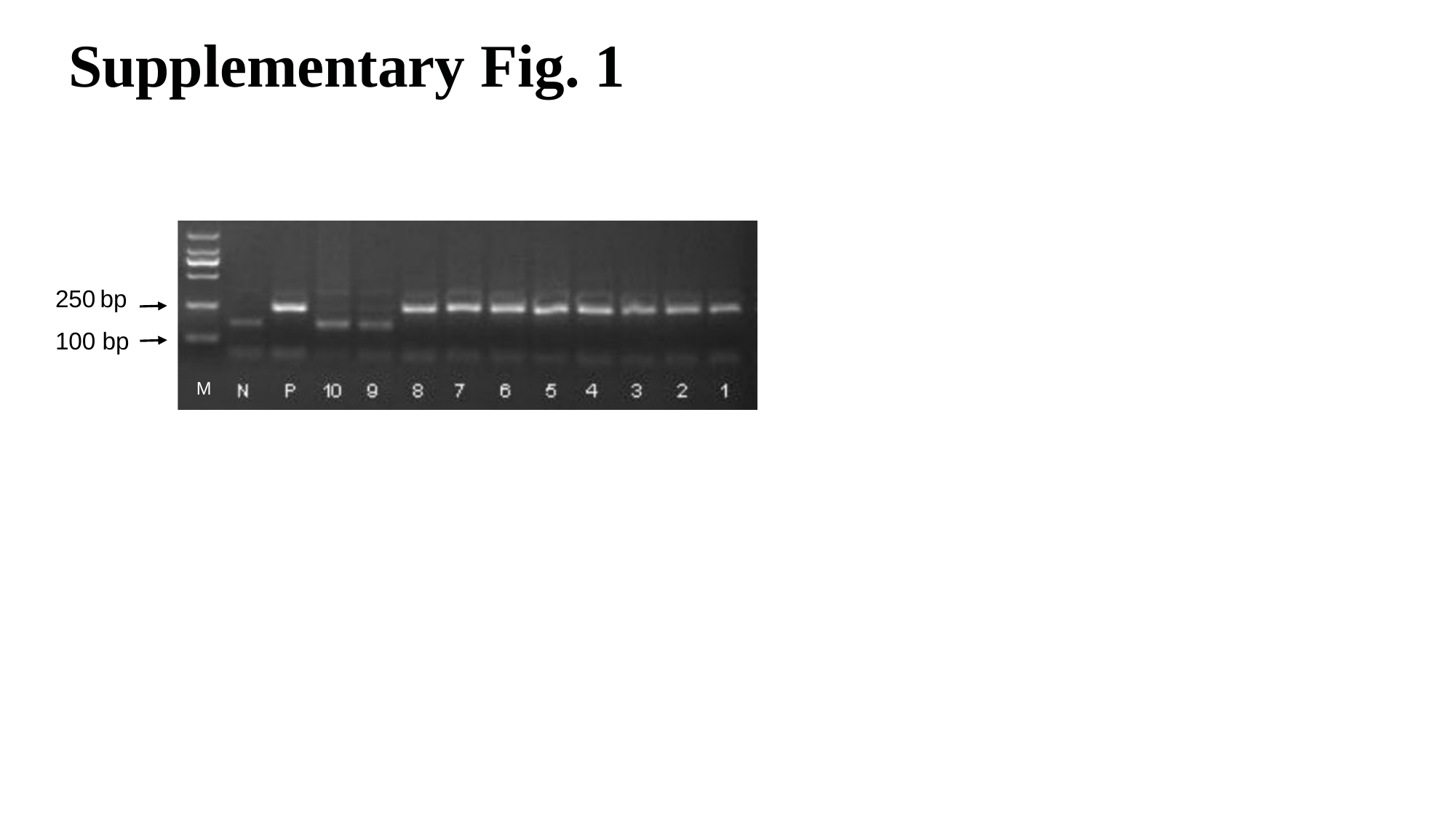

Supplementary Fig. 1
250 bp
100 bp
M

## Slide 2
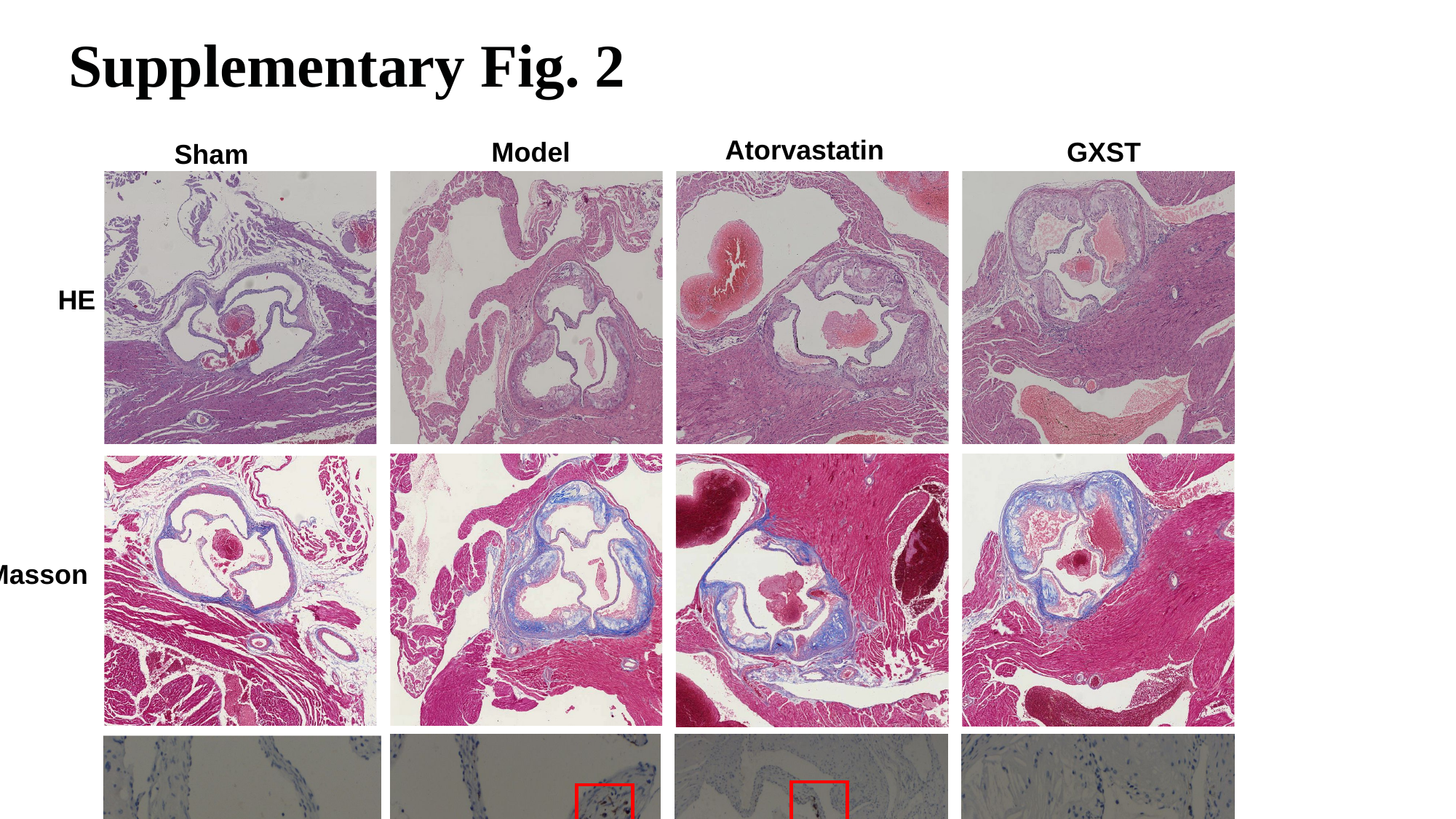

Supplementary Fig. 2
Atorvastatin
Model
GXST
Sham
HE
Masson
CD68

## Slide 3
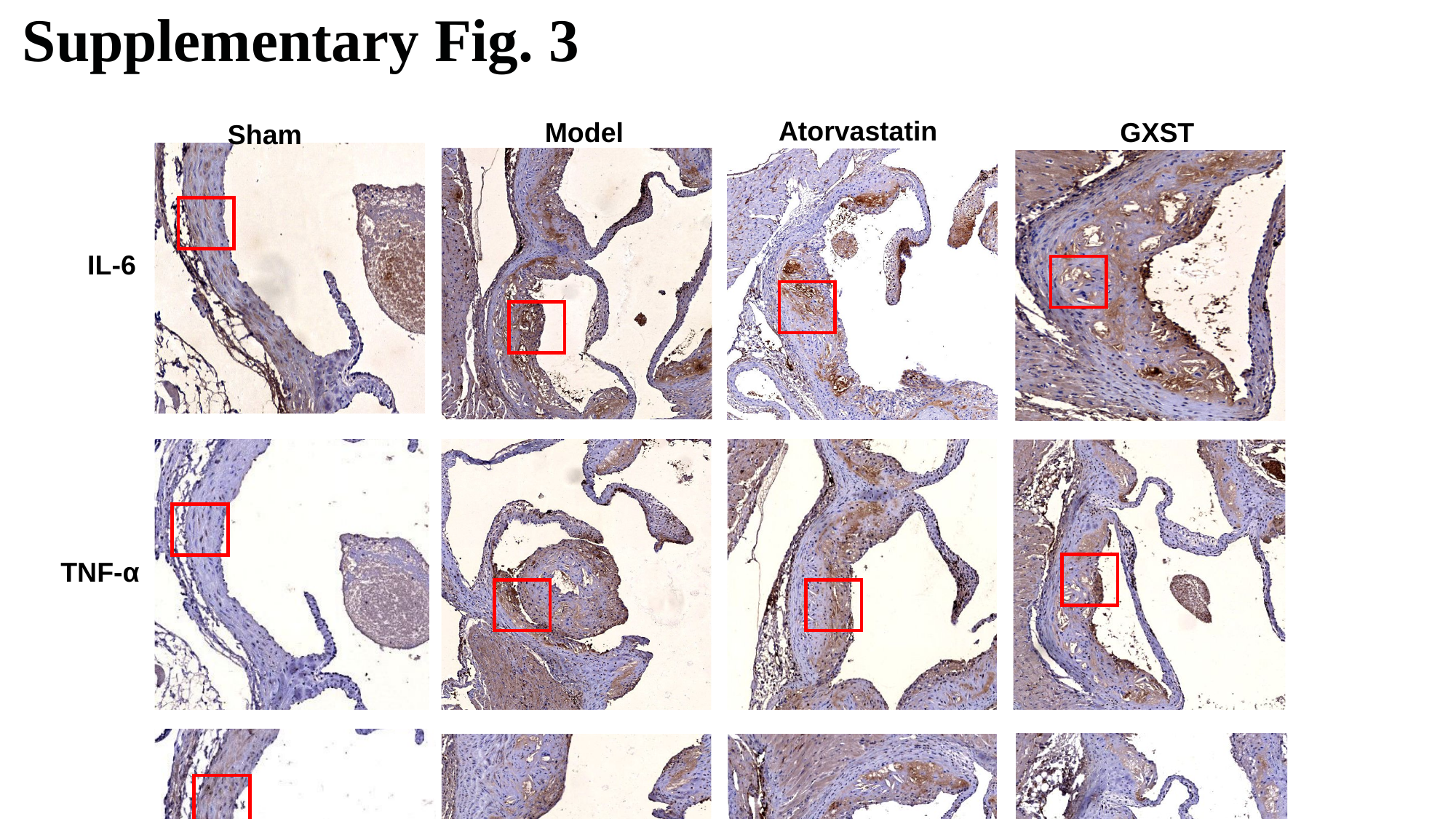

Supplementary Fig. 3
Atorvastatin
Model
GXST
Sham
IL-6
TNF-α
Nrf2
HO-1

## Slide 4
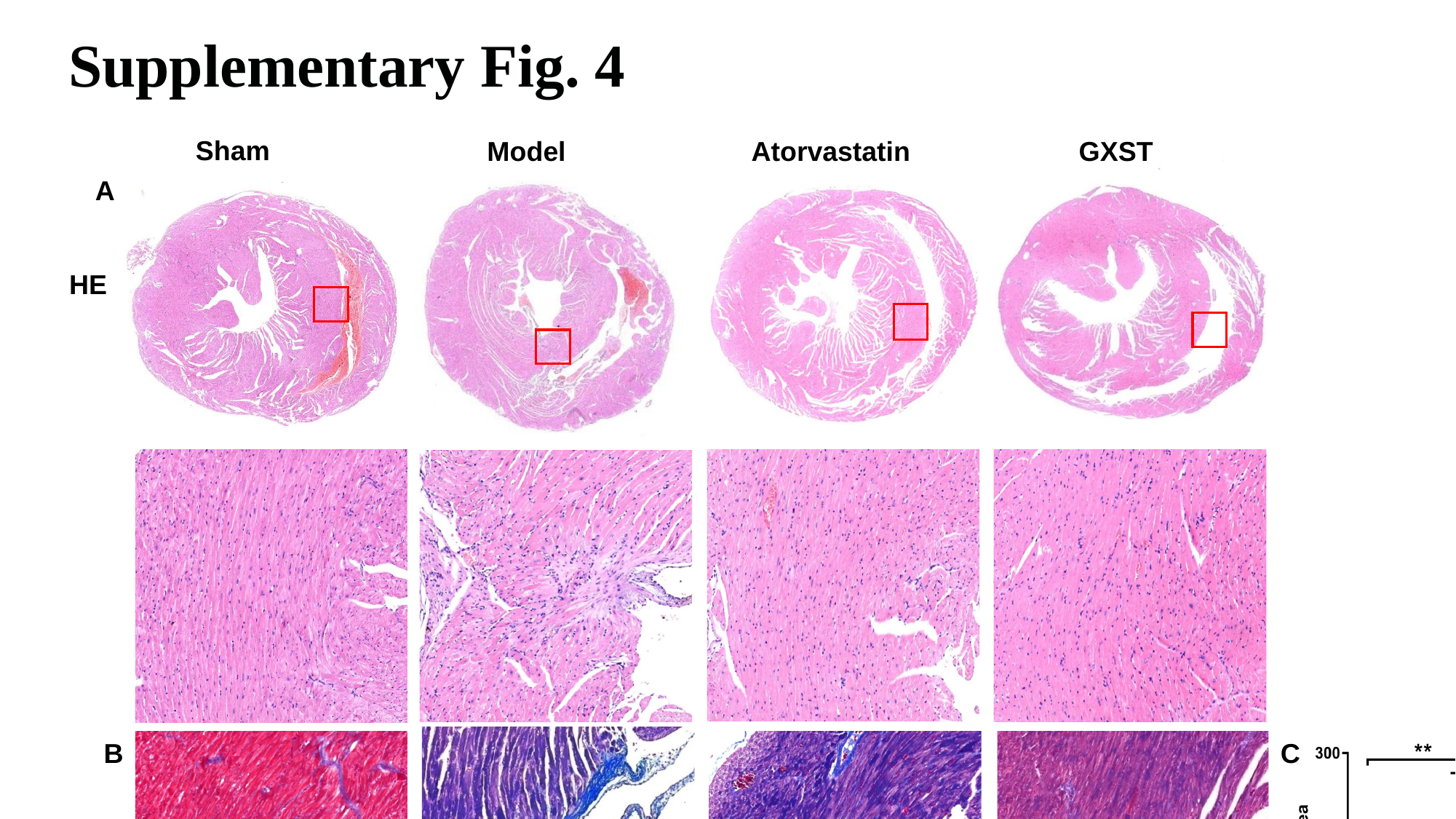

Supplementary Fig. 4
Sham
Model
Atorvastatin
GXST
A
HE
B
C
Masson

## Slide 5
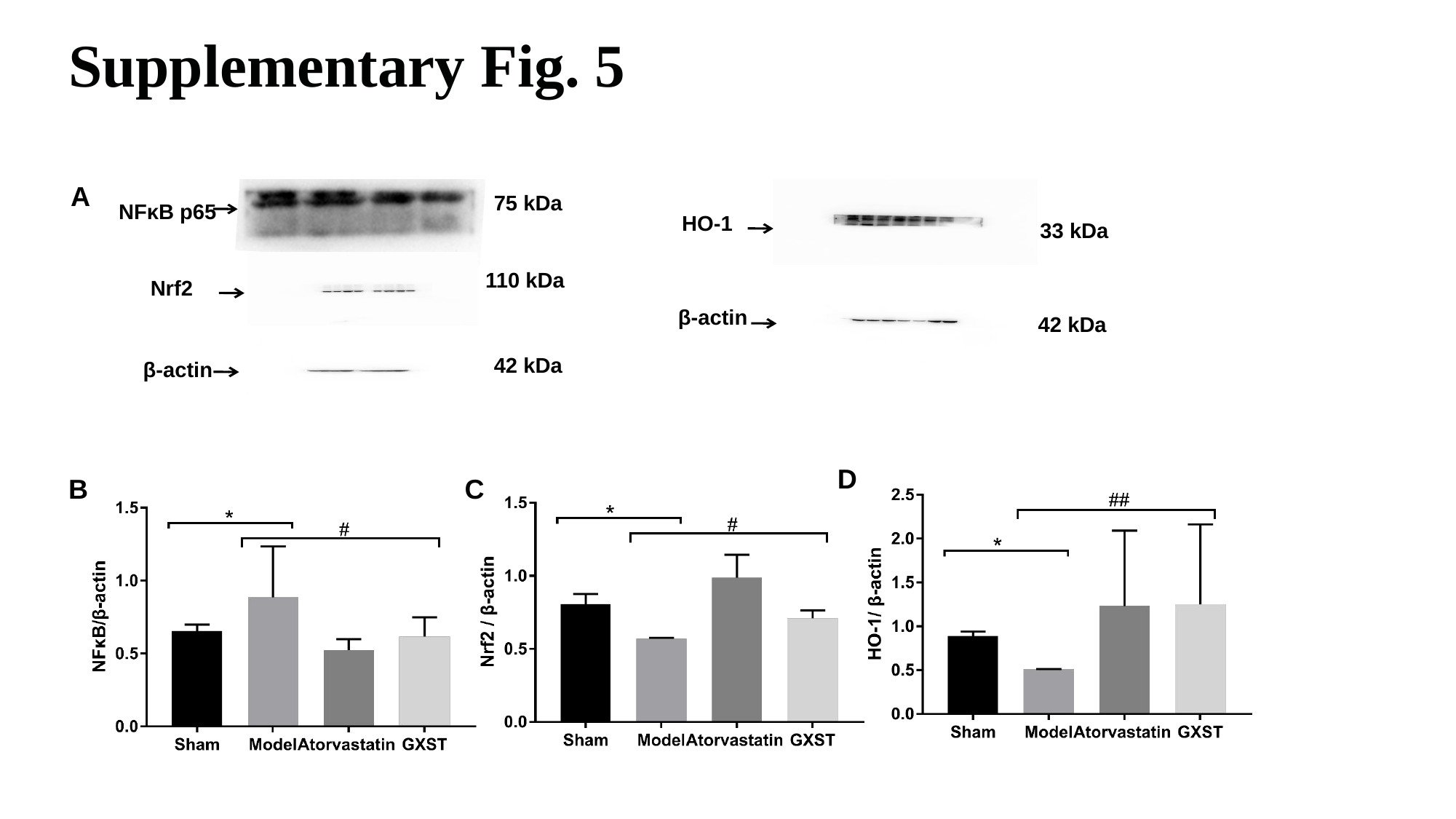

Supplementary Fig. 5
NFκB p65
75 kDa
Nrf2
110 kDa
β-actin
42 kDa
A
HO-1
33 kDa
β-actin
42 kDa
D
B
C
